# Bioherbicidal Evaluation of Methanol Extract of *Sorghum halepense* L. Rhizome and Its Bioactive Components Against Selected Weed Species

**DOI:** 10.3390/molecules30153060

**Published:** 2025-07-22

**Authors:** Jasmina Nestorović Živković, Milica Simonović, Danijela Mišić, Marija Nešić, Vladan Jovanović, Uroš Gašić, Ivana Bjedov, Slavica Dmitrović

**Affiliations:** 1 Institute for Biological Research “Siniša Stanković”, National Institute of the Republic of Serbia, University of Belgrade, Bulevar despota Stefana 142, 11108 Belgrade, Serbia; jasmina.nestorovic@ibiss.bg.ac.rs (J.N.Ž.); dmisic@ibiss.bg.ac.rs (D.M.); 2 Institute of Pesticides and Environmental Protection, Banatska 31b, 11080 Belgrade, Serbia; milica.simonovic@pesting.org.rs (M.S.); vladanjovanovic@pesting.org.rs (V.J.); 3 Faculty of Forestry, University of Belgrade, Kneza Višeslava 1, 11030 Belgrade, Serbia; marija.nesic@sfb.bg.ac.rs

**Keywords:** bioherbicide, Johnson grass, phenolic acid, *Amaranthus retroflexus*, *Datura stramonium*, *Chenopodiastrum murale*, antioxidative enzymes

## Abstract

*Sorghum halepense* (L.) Pers. (common name Johnson grass) is a perennial invasive weed that causes great harm worldwide, and its allelopathy has been demonstrated in a series of experiments. The present study offers new insights into its organ-specific phytochemical profiles using state-of-the-art metabolomic technology and explores the effects of a methanol extract of *S. halepense* rhizomes (*Sh*ER) and its major bioactive compounds (*p*-hydroxybenzoic acid and chlorogenic acid) on three noxious weed species. The phytotoxic effects of *Sh*ER are reflected through the inhibition of seed germination and reduced seedling growth, which are accompanied by changes in the antioxidant system of seedlings. Phytotoxicity is species specific and concentration dependent, and it is more pronounced against *Chenopodiastrum murale* (L.) S. Fuentes, Uotila & Borsch and *Datura stramonium* L. than highly tolerant *Amaranthus retroflexus* L. Catalase (CAT) is most likely the major mediator in the removal of reactive oxygen species, which are generated during germination and early seedling growth of *Ch. murale* exposed to *Sh*ER. The results of the present study imply the high potential of *Sh*ER in the management of amaranthaceous and solanaceous weeds, such as *Ch. murale* and *D. stramonium*, respectively. The present study offers an environmentally friendly solution for the biological control of weeds belonging to the families Amaranthaceae and Solanaceae. Also, the results of this research highlight the possibility of effective management of *S. halepense* by using it as a feedstock for bioherbicide production.

## 1. Introduction

According to the global invasive species database, *Sorghum halepense* (L.) Pers. (Poaceae) is an extremely invasive noxious weed with worldwide distribution [1], which causes significant losses in agriculture and native biodiversity [2,3] and detrimentally affects animals and humans. It is native to the Mediterranean part of Europe, Africa, and Asia [4]. More than a decade ago, this species was marked as a sporadic invasive plant in Serbia [5]. However, recent field surveys indicated that *S. halepense* is aggressively spreading and conquering different habitat types [6,7]. Moreover, it is emerging as a potential menace for agroecosystems in numerous countries across the world [8].

A species’ reproductive strategy significantly impacts its ability to rapidly spread and establish stable populations in a new environment. *S. halepense* has a high asexual and sexual reproductive capacity, and it propagates rapidly by rhizomes and by seeds. Both reproductive strategies are conditioned by environmental factors, such as soil fertility, air temperature, and availability of moisture. An individual *S. halepense* is able to produce as many as 28,000 seeds in a growing season [9]. Additionally, the strong competitive ability of *S. halepense* is conditioned by the synthesis of allelopathic substances (allelochemicals), which can ensure competitive exclusion of other plants in the surroundings [10,11,12,13]. These substances can be synthesized from different plant parts (rhizomes, roots, stem, leaves, and grains). *S. halepense* is reported to be a rich source of allelochemicals, such as benzoic acid, *p*-hydroxybenzoic acid (*p*HBA), vanillic acid, ferulic acid, chlorogenic acid (CHLA), *m*-coumaric acid, *p*-coumaric acid, gallic acid, caffeic acid, *p*-hydroxybenzaldehyde, dhurrin, sorgoleone, *m*-hydroxybenzoic acid, and protocatechuic acid (reviewed in [14]).

To accelerate the search for major allelochemicals in *S. halepense* and explain their modes of action, as well as to assist in designing more effective management strategies for this serious invasive weed, we here provide the comprehensive chemical characterization of methanol-soluble metabolites in different organs (inflorescences, leaves, and rhizomes). It has been revealed that the contents of major phenolic compounds (CHLA, *p*HBA, and *p*-coumaric acid) differ between above ground parts and rhizomes of *S. halepense* [15,16]; however, no previous studies have addressed the organ-specific profiles by adopting an untargeted metabolomic approach. On the other hand, this is an important aspect in the prediction and evaluation of the mutual effects of the metabolites in a given organ and essential information for the development of bioherbicide formulations. Organ-specific chemical differentiation reflects ecological function, as leaves contribute to the aboveground allelopathic effects that suppress neighboring vegetation, while rhizomes mediate belowground interferences with the soil microbiome and root systems of surrounding plants. Not surprisingly, the allelochemicals of *S. halepense* subterranean parts exert allelopathic effects and inhibit important physiological processes in test plants [17]; however, the exact mode of action of the responsible metabolites needs further clarification.

We here explain the effects of a methanol extract of *S. halepense* rhizome (*Sh*ER) and its bioactive components on seed germination and early seedling growth of selected weed species: *Amaranthus retroflexus* L. (common name red-root pigweed, fam. Amaranthaceae)*, Datura stramonium* L. (Jimson weed, fam. Solanaceae), and *Chenopodiastrum murale* (L.) S. Fuentes, Uotila & Borsch (syn. *Chenopodium murale*; nettle-leaved goosefoot, fam. Amaranthaceae). These species are considered to be among the most problematic weed species globally; they are adaptable to different habitat conditions and can cause significant losses in agriculture. Furthermore, they reproduce exclusively by seeds, which are produced in large amounts and retain seed viability for a long time [18,19,20,21]. The dominant method of the suppression of the mentioned weed species is the application of herbicides. However, mechanical control such as hand-pulling or mowing has also proven to be very effective. Optimally, mechanical control measures should be carried out before flowering and should be repeated several times during the vegetative period. Isolated plants or small populations should be hand-pulled, while mowing is recommended for larger infestations. After flowering, appropriate herbicides have to be used in order to control plant spread [6]. Given that the use of chemical herbicides has a negative impact on the environment and on human health, extensive research is being conducted on allelochemicals as potential alternatives for weed management [22,23,24].

Being rich in allelochemicals, *S. halepense* has great potential to be used as a bioherbicide and serve as a basis for the development of new herbicidal formulations. Like other weeds and invasive species, *S. halepense* can be controlled using various approaches, including both mechanical and chemical control methods. Conventional management approaches are limited in their scope to control this weed due to its rapid vegetative growth and increasing herbicidal tolerance [8]. In addition to the timely application of certain measures, proper disposal of mow waste is important in order to prevent further spread of this invasive plant. It is especially important to destroy the mowing residue of flowering plants that have already produced seeds, as well as rhizomes. In Serbia, there are currently no specific collection centers designated for the safe disposal of invasive plant species. Additionally, there is a lack of legislation that defines proper procedures for their removal and disposal.

The aim of this study was to evaluate the bioherbicidal potential of cut rhizomes of *S. halepense* for the development of new bioherbicide formulations, as well as to propose a solution for the safe disposal of this invasive plant. This approach aligns with circular economy principles by converting invasive plant biomass into a value-added product while simultaneously reducing the environmental burden of synthetic herbicides and promoting sustainable weed management practices.

## 2. Results and Discussion

Within the present study, the phytochemical characterization of *S. halepense* rhizomes was conducted in parallel with its phytotoxic evaluation, with the aim of exploring its bioherbicide potential and to predict the benefit of its integration into weed control programs to reduce reliance on conventional herbicides in controlling invasive weeds, such as *D. stramonium* and *A. retroflexus*, and ruderal weed species *Ch. murale*. The weed species analyzed within the present study were selected based on their ecological/phenological preferences, rapid and uniform germination of seeds, and high sensitivity to various natural compounds, chemicals, and environmental conditions [25,26,27]. A study by Yazlik et al. [3], which evaluated the impact of *S. halepense* on species richness, revealed that *A. retroflexus*, *D. stramonium*, and some *Chenopodiastrum* species usually occur in sites characterized by the absence of *S. halepense*, which indicated possible allopathic interactions. On the other hand, allelopathic potential against a number of weeds was also recorded for *A. retroflexus* [28,29], *D. stramonium* [30,31,32], and *Ch. murale* [33,34,35]. *A. retroflexus* is a facultative short-day plant, characterized by the production of a high number of non-dormant seeds [36]. It is native to America, but due to its strong adaptability and reproductive ability, *A. retroflexus* is distributed worldwide, mainly in soybean, corn, wheat, sweet potato, and cotton fields [37]. *A*. *retroflexus* has become a malignant weed that competes with crops for nutrients and water, resulting in significant decreases in grain yield and quality of crops [38]. *D. stramonium* is a hallucinogenic plant with both poisonous and medicinal properties. It is native to deserts of the North American Southwest, Central and South America, Europe, Asia, and Africa. It is a problematic weed present in nearly 100 countries, disrupting the cultivation of over 40 different crops and showing resistance to triazine herbicides [39]. Each plant produces between 1500 and over 30,000 seeds, which are spread through water, farm machinery, or as contaminants in crop seeds, hay, and feed grains. These seeds can remain viable in the soil for many decades. *Ch. murale* is a widespread annual weed species with significant economic impact [40]. Its distinct biological traits—such as high reproductive ability, seed dormancy, strong persistence in the soil seed bank, capacity to germinate and grow under varied environmental conditions and abiotic stresses, and production of allelopathic compounds—enable it to infest a wide range of cropping systems. It influences negatively the growth of crops and decreases biological nitrogen fixation in legumes [20].

Generally, the integration of multiple management practices can be an effective way to control these weed species but also to minimize the risk of herbicide resistance. Understanding the physiological responses of *A. retroflexus*, *D. stramonium*, and *Ch. murale* to the allelopathic action of natural products can assist in developing more effective control methods. Thus, our aim was to examine the phytotoxic abilities of *S. halepense* against these weed species.

### 2.1. Untargeted Metabolomics of Sorghum halepense Reveals Organ-Specific Metabolite Profiles

Initially, the chemical composition of methanol extracts of *S. halepense* inflorescences, leaves, and rhizomes was characterized. UHPLC-QTOF-MS analysis resulted in the identification of a total of 60 compounds that belonged to hydroxybenzoic (13 compounds) and hydroxycinnamic (21 compounds) acid derivatives, flavonoid glycosides (11 compounds), flavonoid aglycones (5 compounds), fatty acids (8 compounds), and lignans (2 compounds). The peak numbers, compound names, retention times, molecular formulas, calculated and exact masses, mass accuracy errors, major MS^2^ fragment ions, as well as the presence of certain metabolites in different parts of *Sorghum halepense* are summarized in Table 1. In addition, in the same table, references related to the previous identification of these compounds in different *Sorghum* species or other species from the family Poaceae are listed. Table S1 summarizes the peak areas of the identified compounds in the investigated extracts analyzed in three biological replicates.

Among hydroxybenzoic acids, hexosyl derivatives were especially abundant, giving specific fragments resulting from the loss of 162 Da (Table 1). The analyzed organs of *S. halepense* (inflorescences, leaves, and rhizomes) shared similar profiles of hydroxybenzoic acids.

The highest portion of identified phenolic acid derivatives belonged to the group of hydroxycinnamic acids, which were mainly represented by esters with quinic acid and glycerol (Table 1). Two non-specific compounds (**18** and **19**) for the investigated plant were found in all samples. By studying the fragmentation of compound **19**, it was concluded that it gave specific fragments of caffeoylquinic acid and also contained hexose and sinapic acid residues. The proposed structure and detailed fragmentation of this compound are shown in Figure S1. In addition to the exact mass of the molecular ion, the MS^2^ fragments matched the exact masses of the proposed structures.

The results revealed the presence of 11 flavonoid glycosides belonging mainly to the subgroup of flavones (derivatives of apigenin, luteolin, eriodictyol, chrysoerol, and tricin) and flavonols (quercetin derivatives) (Table 1). Two compounds (**37** and **42**), present in all analyzed samples, were identified as flavonoid *C*-glycosides, with the specific generation of fragment ions resulting from the loss of 120 and/or 90 Da, while the other 9 compounds were flavonoid *O*-glycosides. Compound **43**, which was identified only in sorghum flowers, was not previously reported in this plant species, but its fragmentation (Figure S3) was consistent with available literature data [61]. Similarly, compound **44**, detected only in *S. halepense* leaf extract, had not been previously reported in *Sorghum* species, nor in any other species from the family Poaceae. The proposed structure and detailed fragmentation of this compound, shown in Figure S4, was in accordance with the literature [62]. Among the analyzed plant organs, flavonoid glycosides were the most abundant in inflorescences, followed by leaves. In roots, only two compounds belonging to this group of flavonoids were detected (**37** and **42**).

In the case of flavonoid aglycones and fatty acid derivatives, all identified compounds were previously reported for the genus *Sorghum* [44,51]. The qualitative composition of flavonoid aglycones was significantly different between *S. halepense* organs, and this group of flavonoids was the most abundant in rhizomes (Table 1). Fatty acids were especially abundant in inflorescences and rhizomes.

Two lignan derivatives (**59** and **60**), which were present only in *S. halepense* rhizomes, with a molecular ion at 727 *m*/*z*, were identified as oryzativol A and oryzativol B. These two isomeric compounds gave an MS^2^ base peak corresponding to deprotonated coumaric acid (163 *m*/*z*), as well as fragments resulting from the fragmentation of coumaric acid (119 and 145 *m*/*z*). These compounds are named after rice (*Oryza sativa* L.), from which they were first isolated [58].

To study the interrelation between the expression profiles of identified metabolites in different *S. halepense* organs, the metabolomic data (peak areas) were analyzed using two unsupervised statistical methods: principal component analysis (PCA) and hierarchical cluster analysis (HCA).

**Figure 1:**
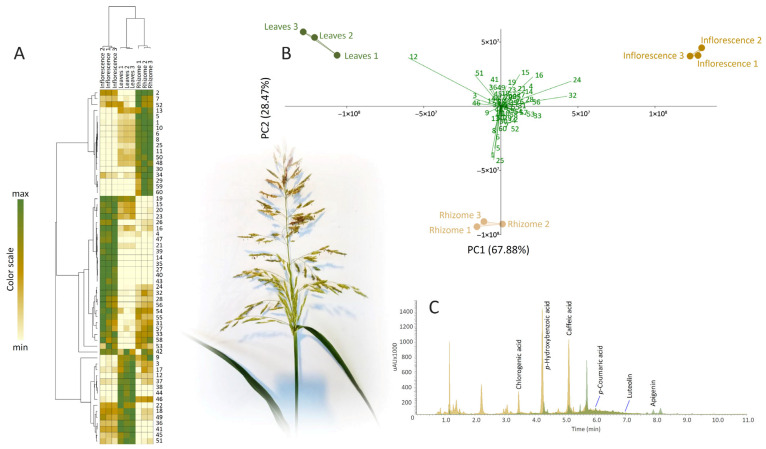
Methanol extracts of *S. halepense* inflorescences, leaves, and rhizomes were subjected to non-targeted and targeted metabolic profiling and further to chemometric data analysis. (**A**) Heatmap of the scaled LC-ESI-QTOF-MS/MS data with the samples and compounds arranged according to the HCA, adopting the Pearsons’s method of cluster agglomeration. The values (peak areas) are scaled between min and max for each raw independently, as indicated at the color scale. Numbers of compounds correspond to those from Table 1. (**B**). PCA biplot constructed based on the LC-ESI-QTOF-MS/MS data, with the first two PCs explaining 67.88% and 28.47% of the variability, respectively; (**C**) Representative UHPLC/DAD chromatograms of *S. halepense* rhizomes recorded at λ_max_ = 210 (yellow line) and 320 nm (green line).

HCA clearly depicted the linkages between inflorescences, leaves, and roots of *S. halepense*. Two major clusters were distinguished, one comprising leaves and rhizomes, while inflorescences formed a separate cluster, which indicated that the rhizomes and leaves were phytochemically more similar to each other than to inflorescences. On the other hand, two major clusters of metabolites were visible in the HCA matrix. The first cluster (A) contained metabolites predominating in the leaves of *S. halepense*. The second cluster (B) could be divided into two subclusters based on the differential expression of metabolites in inflorescences (b1) and rhizomes (b2) (Figure 1A,B).

PCA revealed that PC1 and PC2 cumulatively explained 96.35% of the total variance. Samples of inflorescences were separated from those of leaves and roots along PC1, explaining 67.88% of the total variation. On the other hand, inflorescences and leaves segregated from rhizomes along PC2, which explained 28.47% of the variability. The major contributors to PC1 were 1-*O*-coumaroyl-glycerol (**24**) and 1,3-*O*-coumaroyl-feruloyl-glycerol (**32**), which were the most abundant in inflorescences, but also dihydroxybenzoic acid 2 (**12**), predominating in leaves. Along PC2, samples were distinguished mainly by compounds dihydroxybenzoic acid 2 (**12**), trihydroxyoctadecadienoic acid 1 (**51**), 3-*O*-caffeoylquinic acid 2 (**15**), and 5-*O*-caffeoylquinic acid (**16**), which were abundant in above-ground parts. Compounds abundant in rhizomes, *p*-coumaric acid (**25**) and hydroxybenzoyl hexoside 1 and 2 (**1** and **5**, respectively), were also significant contributors to the diversification along PC2.

The combination of HCA and PCA offered a glimpse of the possible usefulness of the identified metabolites for phytochemical differentiation of the three organs of *S. halepense*. Six compounds were exclusively present in inflorescences, four belonging to the group of flavonoid glycosides (**35**, **39**, **40**, **43**) and two hydroxycinnamic acid derivatives (**14**, **27**). Two derivatives of hydroxycinnamic acid (**29** and **30**) and the two lignan derivatives (**59** and **60**) were recorded only in rhizomes, while leaves were characterized by the presence of two luteolin derivatives (**38** and **44**), which were not recorded in other analyzed organs. Hydroxybenzoic acid derivatives were especially abundant in rhizomes of *S. halepense*, as well as flavonoid aglycones apigenin and chrysoerol (**48** and **50**, respectively) and *p*-coumaric acid (**25**), belonging to the group of hydroxycinnamic acid derivatives.

### 2.2. pHBA and CHLA Are the Major Phenolics of S. halepense Rhizomes

Rhizomes, which represent subterranean stems that are a key link between the morphology and ecology of *S. halepense*, were especially interesting for their phytochemical composition. This part of the plant is directly involved in plant–biotic interactions in the rhizosphere and is most likely responsible for the phytotoxic and allelopathic effects of highly invasive *S. halepense* against surrounding plants. The dynamic root exudation of phenolic allelochemicals from Johnson grass roots has been proposed as a mechanism to promote the invasive success of the plant [63]. The temporal and spatial dynamics of the release of these compounds are likely to be a key component of their toxicity to neighboring plants [64]. Other key attributes in the invasion success of *S. halepense* most likely include herbicide tolerance, diverse propagation mechanisms, rapid development, and strong competitiveness. Previous studies showed inhibitory effects of the extracts of rhizomes, rhizosphere soil, and soil incorporating decaying rhizomes, as well as leaves of Johnson grass, on the root growth of various tested crops [15,16,17,63,65,66,67]. The phytotoxic and antimicrobial activities of major *S. halepense* bioactive constituents, including phloroglucinol, CHLA, *p*-hydroxybenzyl alcohol, *p*-coumaric acid, *p*-hydroxybenzaldehyde, *p*HBA, ethyl *p*-hydroxybenzoate, tricin, diosmetin, luteolin, apigenin, dhurrin, and taxiphyllin, have been documented [15,16,63].

Abdul-Wahab and Rice [15] identified CHLA, *p*-coumaric acid, and *p*-hydroxybenzaldehyde as the major phytotoxic compounds in extracts from wild sorghum leaves and rhizomes. Shah et al. [68] also reported that *S. halepense* rhizomes are a rich source of *p*HBA, CHLA, and *p*-coumaric acid, among other identified compounds. This is consistent with our results of the UHPLC/DAD/(–)HESI–MS^2^ quantification of the major phenolics in rhizomes, as *p*HBA, CHLA, and *p*-coumaric acid were identified as the dominant phenolic acids in the methanol extract of wild sorghum rhizomes (Table 1). The representative DAD chromatograms at λ = 260 and 320 nm are shown in Figure 1C. *p*HBA reached 3023.01 ng mg^−1^ dry weight (DW) of rhizome, and the second most abundant compound was CHLA (338.61 ng mg^−1^ DW). The concentration of *p*-coumaric acid was 113.28 ng mg^−1^ DW. Among flavonoids, luteolin (2.056 ng mg^−1^ DW) and apigenin (0.23 ng mg^−1^ DW) were the most abundant constituents in *S. halepense* rhizomes.

### 2.3. pHBA and CHLA Are Drivers of S. halepense Phytotoxicity at the Germination Stage of A. retroflexus, D. rtramonium, and Ch. murale Seeds

**Figure 2:**
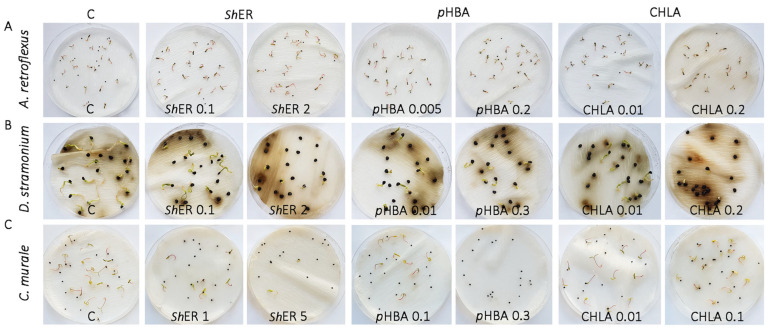
Bioherbicidal potential of *Sh*ER (0.1–5 mg mL⁻^1^), *p*HBA, and CHLA (0.005–0.3 mg mL⁻^1^) against seed germination and seedling growth of (**A**) *A. retroflexus*, (**B**) *D. stramonium*, and (**C**) *Ch. murale* at 7, 10, and 11 days after treatment, respectively.

**Figure 3:**
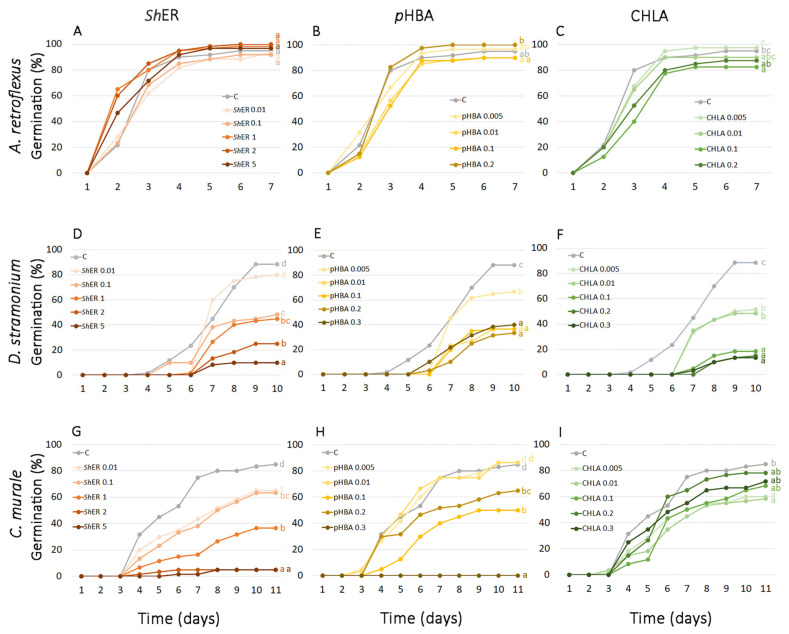
Seed germination dynamics of *A. retroflexus*, *D. stramonium*, and *Ch. murale* after treatment with (**A**,**D**,**G**, respectively) *Sh*ER (0.01–5 mg mL⁻^1^), (**B**,**E**,**H**, respectively) *p*HBA, and (**C**,**F**,**I**, respectively) CHLA (0.005–0.3 mg mL⁻^1^). Control treatment (C) was performed using distilled water. The values with the same letter indicate statistically homogeneous groups (*p* ≤ 0.05), according to Fisher’s LSD test, and refers to the data recorded on the last day of the experiment.

The first records of germination in non-treated *A. retroflexus* were observed 2 days after the start of the treatment (DT). The maximum germination percentage was reached at 5 DT (95%). The total germination percentage at 7 DT was not significantly influenced by any treatment with *Sh*ER, and it ranged between 92% and 100%. Germination dynamics were slightly inhibited during early phases of the experiment (until the second day) by treatment with 0.01 mg mL^−1^ *Sh*ER (inhibition of 2% and 4%, respectively), while higher *Sh*ER concentrations (from 1 to 5 mg mL^−1^) caused even slight stimulation of seed germination (from 2% to 5%) (Figure 2 and Figure 3A). The effect of *p*HBA (0.005, 0.01, 0.1, and 0.2 mg mL^−1^) on the final germination of *A. retroflexus* at 7 DT was not significantly different from the control, where a 95% germination rate was recorded. The lowest and highest applied *p*HBA concentrations (0.001 and 0.2 mg mL^−1^) slightly stimulated final germination by 2% and 5%, respectively, while 0.01 and 0.1 mg mL^−1^ slightly inhibited final germination (5% each) (Figure 2 and Figure 3B). CHLA displayed a stronger inhibitory effect on the final germination of *A. retroflexus* seeds than *p*HBA. Treatment with 0.1 mg mL^−1^ CHLA resulted in a statistically significant inhibition (13%) compared to the control. The lowest CHLA concentration (0.001 mg mL^−1^) induced a slight stimulation of final germination (3%) (Figure 2 and Figure 3C).

At 10 DT, *D. stramonium* seeds reached a final germination of 88%. In all treatments with *Sh*ER (0.1, 1, 2 and 5 mg mL^−1^), with the exception of 0.01 mg ml^−1^ *Sh*ER, there was a statistically significant inhibition of germination compared to the control (from 45 to 89%), (*p* ≤ 0.05) (Figure 2 and Figure 3D). A similar trend of inhibition of *D. stramonium* seed germination was observed for *p*HBA and CHLA treatments (0.005, 0.01, 0.1, 0.2, and 0.3 mg mL^−1^). For both phenolic acids tested, inhibition of germination was statistically significant for all treatments compared to the control. At the highest concentrations applied (0.3 mg mL^−1^), CHLA had a stronger inhibitory effect (85%) than *p*HBA (55%) (Figure 2 and Figure 3E,F).

The final germination of *Ch. murale* seeds at 11 DT reached 85% in the control. The treatment of seeds with *Sh*ER at concentrations of 0.01, 0.1, and 1 mg mL^−1^ resulted in the inhibition of germination (24%, 25%, and 57%, respectively), while at 2 and 5 mg mL^−1^ *Sh*ER, germination was almost completely inhibited (94%, both). A statistically significant reduction in germination occurred at all *Sh*ER treatments when compared to the control (Figure 2 and Figure 3G). The effects of two dominant phenolic acids (CHLA and *p*HBA) at different concentrations (0.005, 0.01, 0.1, 0.2, and 0.3 mg mL^−1^) on the germination of *Ch. murale* seeds were also studied. At 11 DT, the final germination of *Ch. murale* seeds was slightly increased (2% each) at the lower applied concentrations of *p*HBA (0.005 and 0.01 mg/L), while treatments with 0.1 and 0.2 mg mL^−1^ *p*HBA showed a statistically significant inhibition of *Ch. murale* germination from 41% to 24%, respectively. Complete inhibition of *Ch. murale* germination occurred at the treatment with the highest *p*HBA concentration (0.3 mg mL^−1^) (Figure 2 and Figure 3H). This result was in agreement with Reigosa et al. [69], who reported that phenolic acids structurally related to *p*HBA, ferulic acid and vanillic acid, inhibited the germination of *Chenopodium album* L. at high concentrations (10 mM, both). The final germination of *Ch. murale* after 11 DT was lower than the control at all CHLA concentrations applied (0.005, 0.01, 0.1, 0.2, and 0.3 mg mL^−1^), while a statistically significant inhibition occurred at 0.005 and 0.01 mg mL^−1^ CHLA, where inhibition was 29% and 31%, respectively (Figure 2 and Figure 3I). Our results demonstrated the prominent phytotoxic effect of *Sh*ER at all applied concentrations (0.01, 0.1, 1, 2, and 5 mg mL^−1^) against *D. stramonium* and *Ch. murale*, which was reflected through the marked inhibition and slowed dynamics of seed germination. This effect was concentration dependent. *p*HBA and CHLA were significant contributors to the phytotoxic effects of *Sh*ER, as the treatments with these phenolic acids showed very similar trends of germination inhibition, which was concentration dependent (Figure 2 and Figure 3). Interestingly, *Sh*ER displayed more severe effects than *p*HBA and CHLA, suggesting that other metabolites, and most likely their synergistic action, were important for the overall phytotoxicity, which will be the subject of our further investigations. The germination of *A. retroflexus* seeds was not significantly affected by *Sh*ER, *p*HBA, and CHLA. The exception was the treatment with 0.1 mg mL^−1^ CHLA, where a slight inhibition of seed germination was recorded (Figure 2 and Figure 3).

Previous studies showed the strong allelopathic effect of an aqueous extract of *S. halepense* aerial parts that suppressed various weeds, including *A. retroflexus* [70]. The inhibitory effect of *S. halepense* aqueous extract on *A. retroflexus* seed germination was organ and concentration dependent [71]. To the best of our knowledge, there are no previous literature data on the allelopathic effects of *S. halepense* rhizomes on *D. stramonium* and *Ch. murale* seed germination.

### 2.4. Methanol Extract of S. halepense Rhizome and Its Major Constituents, pHBA and CHLA, Affect Early Seedling Growth of A. retroflexus, D. stramonium, and Ch. murale

**Figure 4:**
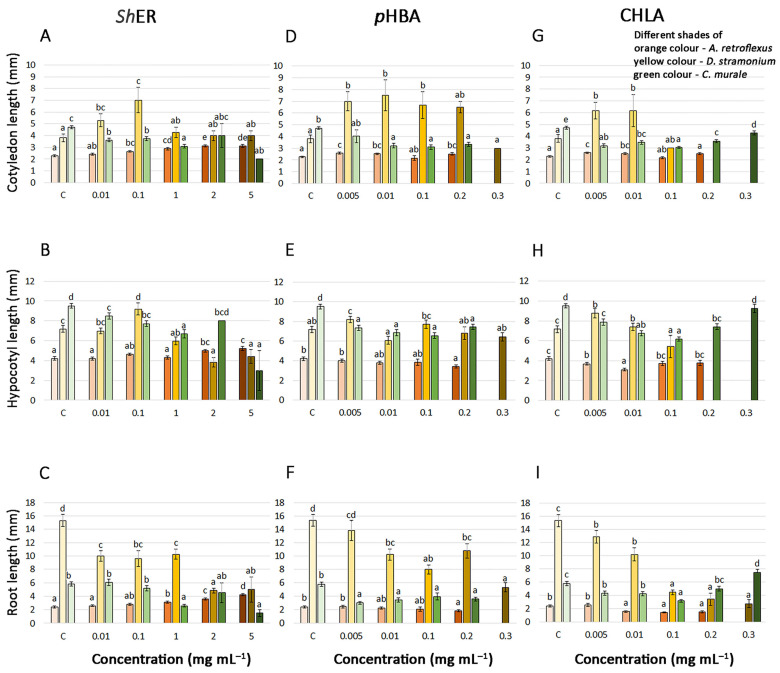
Effects of various concentrations of *Sh*ER (0.01–5 mg mL⁻^1^), *p*HBA, and CHLA (0.005–0.3 mg mL⁻^1^) on (**A**,**D**,**G**, respectively) cotyledon, (**B**,**E**,**H**, respectively) hypocotyl, and (**C**,**F**,**I**) root growth of *A. retroflexus*, *D. stramonium*, and *Ch. murale* seedlings at 7, 10, and 11 days after treatment, respectively. Control treatment (C) was performed using distilled water. The values with the same letter indicate statistically homogeneous groups (*p* ≤ 0.05), according to Fisher’s LSD test.

The phytotoxic effects of different *Sh*ER concentrations, and of *p*HBA and CHLA, on *A. retroflexus*, *D. stramonium*, and *Ch. murale* seedling growth at 7, 10 and 11 DT, respectively, were studied by recording cotyledon, hypocotyl, and root length (Figure 4). The results showed that the analyzed weed species displayed differential sensitivity to *Sh*ER in terms of early seedling growth.

*Sh*ER was not phytotoxic against *A. retroflexus* seedlings, and high concentrations even increased cotyledon (from 0.1 to 5 mg mL^−1^), hypocotyl (2 and 5 mg mL^−1^), and root length (from 1 to 5 mg mL^−1^) with respect to the non-treated seedlings (Figure 2 and Figure 4A–C). When the effects of *p*HBA were analyzed, it was observed that treatments with 0.005, 0.01, and 0.2 mg mL^−1^ of this phenolic acid slightly increased the length of *A. retroflexus* cotyledons (Figure 4D). Inhibitory effects on the hypocotyl and root growth of *A. retroflexus* seedlings were visible only for the 0.2 mg mL^−1^ *p*HBA treatment (Figure 4E,F). The cotyledon length of *A. retroflexus* seedlings was slightly stimulated in the presence of 0.005 and 0.01 mg mL^−1^ CHLA, while hypocotyl and root growth were inhibited at CHLA concentrations higher than 0.01 and 0.005 mg mL^−1^ CHLA, respectively (Figure 4H,I).

Lower applied *Sh*ER concentrations (0.01 and 0.1 mg mL^−1^) induced cotyledon elongation in *D. stramonium* seedlings at 10 DT, while higher concentrations had no significant effect (Figure 4A). Stimulation of hypocotyl growth was recorded for the treatment with 0.1 mg mL^−1^ *Sh*ER, while higher concentrations reduced the length of hypocotyls in *D. stramonium* seedlings (Figure 4B). All applied *Sh*ER concentrations reduced the length of *D. stramonium* roots, and the effect was the most pronounced for treatments with 2 and 5 mg mL^−1^ *Sh*ER (Figure 4C). Concentrations of *p*HBA in the range from 0.005 to 0.1 mg mL^−1^ significantly stimulated the length of *D. stramonium* cotyledons (Figure 4D). The length of *D. stramonium* hypocotyls was not affected by the *p*HBA treatments, with the exception of 0.005 mg mL^−1^, which was stimulatory (Figure 4E). Interestingly, low concentrations of CHLA (0.005 and 0.01 mg mL^−1^) stimulated the length of *D. stramonium* cotyledons (Figure 4G). Treatments with 0.1 mg mL^−1^ CHLA and higher significantly reduced *D. stramonium* hypocotyl length (Figure 4H). The root length of *D. stramonium* decreased with increasing CHLA concentration, and this effect was especially pronounced for treatments with CHLA from 0.1 to 0.3 mg mL^−1^ (Figure 4I).

In *Ch. murale* seedlings, treatment with *Sh*ER resulted in reductions in cotyledon and hypocotyl lengths at all applied concentrations when compared to the non-treated seedlings (Figure 4A,B). The root length of *Ch. murale* seedlings was also reduced for treatments with *Sh*ER from 1 to 5 mg mL^−1^ (Figure 4C). The effect of *p*HBA on *Ch. murale* cotyledon and hypocotyl lengths was reflected by slight reductions for treatments with concentrations higher than 0.01 and 0.005 mg mL^−1^, respectively (Figure 4D,E). Root growth was reduced for treatments with *p*HBA concentrations higher than 0.005 mg mL^−1^ (Figure 4F). The effect of CHLA on *Ch. murale* seedling growth was visible through the inhibition of cotyledon growth for all treatments, and in reductions in hypocotyl length when applying 0.005 to 0.2 mg mL^−1^ of this phenolic acid (Figure 4G,H). Treatment with 0.3 mg mL^−1^ CHLA stimulated the root length of *Ch. murale* seedlings (Figure 4I).

Allelopathic substances usually affect early seedling growth and development more than the seed germination process, as germination is more dependent on the reserves in seeds and is less sensitive to exogenous factors [72]. Overall, it appeared that the roots of the tested weeds were more sensitive to the effects of the allelochemicals present in *Sh*ER than the hypocotyls and cotyledons. It is well documented that phenolic allelochemicals inhibit plant cell division and root growth and may also cause changes in cell ultrastructure and affect the permeability of the cell membrane, thus affecting the overall growth and development of plants [73]. Phenolic allelochemicals have been shown to affect plant respiration by reducing oxygen uptake capacity, while the effects on photosynthesis are mainly reflected in reductions in chlorophyll content and photosynthetic rate [73]. They can also reduce or activate the physiological function of plant hormones, such as indole-3-acetic acid, gibberellic acid, cytokinin, ethylene, and abscisic acid, which, in turn, can inhibit normal physiological plant growth [74]. Some phenols (ferulic acid and cinnamic acid) can inhibit protein synthesis or amino acid transport [75], which substantially affects the growth of plants. In addition, phenolic allelochemicals can prevent plants from absorbing nutrients from the environment, thus affecting normal plant growth [73].

In *Trifolium alexandrinum* L., wild sorghum exudates stimulated the growth of beneficial nitrogen-fixing bacteria from the genus *Rhizobia* sp., while in *Vicia faba* L., they activated the growth of some *Bacillus* bacteria responsible for the inhibition of the growth of parasitic *Orobanche* plants [76].

Allelopathic effects are species specific, and plant organs are differentially sensitive to the actions of allelochemicals [77,78,79]. Previous studies showed that an extract of *S. bicolor* rhizomes inhibited the growth of roots in rice seedlings [80], while exudates of roots affected the cell cycle in beans and reduced the number of cells during cell division [81]. Asgharipour and Armin [82] demonstrated that extracts from the roots and leaves of wild sorghum inhibited the seed germination and early seedling growth of medicinally important plants, such as *Plantago ovata* Forssk., *P. indica* L., *Foeniculum vulgare* Mill., and *Ocimum basilicum* L. Parks and Rice [83] found that the growth of some soil algae was inhibited by a rhizome extract of wild sorghum. They suggested that toxins released by plants may affect other higher plants by affecting the soil microflora. A cyanogenic glycoside, taxiphyllin, was isolated from a methanol extract of wild sorghum rhizomes, which, together with other phytotoxic compounds, inhibited the growth of tomato and radish seedling roots, as well as the growth of some bacteria, while sorghum rhizome exudates did not affect the root growth of tomato and radish seedlings but did affect bacterial growth and slowed germination in 3 out of 5 weed species tested [16].

### 2.5. Methanol Extract of S. halepense Rhizome, pHBA, and CHLA Modulate the Activities of Antioxidant Enzymes in Ch. murale Seedlings

Phenolic allelochemicals enter through the cell membrane of plants and alter the activity and function of certain enzymes. Previous results showed that CHLA, caffeic acid, and catechol can inhibit phosphorylase activity, cinnamic acid and its derivatives inhibit the hydrolysis of ATPase, while tannic acids can inhibit the activities of peroxidase (POX), CAT, and cellulase [84]. It is well known that exposure to phytotoxic compounds may result in excessive production and accumulation of reactive oxygen species (ROS) in plants, which further activates antioxidant defense responses [85].

**Figure 5:**
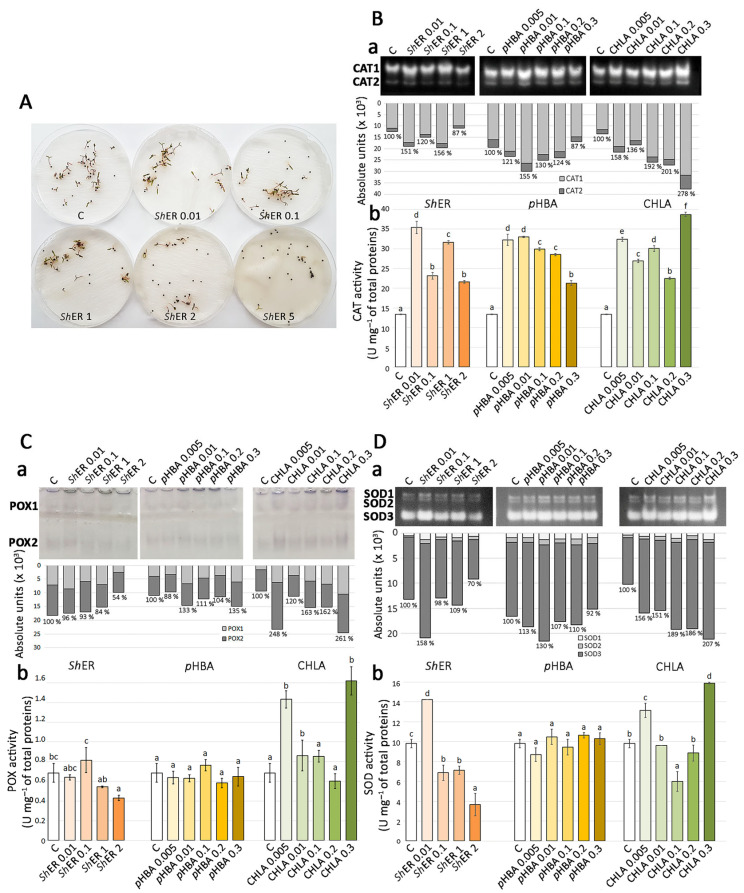
(**A**) Enzyme activity analyzed in *Ch. murale* seedlings after treatment with *Sh*ER for 11 days. Native PAGE (**a**) and spectrophotometric quantification (**b**) of (**B**) CAT, (**C**) POX, and (**D**) SOD activities were performed in *Ch. murale* seedlings after treatment with *Sh*ER (0.01–2 mg mL⁻^1^), CHLA, and *p*HBA (0.005–0.3 mg mL⁻^1^). Control treatment (C) was performed using distilled water. The values with the same letter indicate statistically homogeneous groups (*p* ≤ 0.05), according to Fisher’s LSD test.

Being extremely sensitive to the allelopathic effects of *Sh*ER, *p*HBA, and CHLA, *Ch. murale* was chosen as the model species to analyze the effects of the alleopathic agents on its antioxidant system. The aim was to investigate whether and in what way *Sh*ER and its dominant phenolic constituents, CHLA and *p*HBA, affect the oxidative status of 11-day-old *Ch. murale* seedlings (Figure 5A). The changes in the activities of CAT, POX, and superoxide dismutase (SOD) were evaluated using two methods: electrophoretic separation of their isoforms by native polyacrylamide gel electrophoresis (native PAGE), followed by specific staining assays to visualize the enzyme activities in the gel, and spectrophotometric quantification of the total CAT, POX, and SOD activities (Figure 5).

As previously described [86], two isoforms of CAT (CAT1 and CAT2) were clearly observed in *Ch. murale* seedlings on the gel (Figure 5B(a)). The activity of CAT1 isoform was found to be higher than that of CAT2 isoform. The activity of total CATs in the control groups is shown as 100%. The activity of total CAT increased for treatments with 0.01–1 mg mL^−1^ *Sh*ER with respect to the control (Figure 5B(a)). Treatment with 2 mg mL^−1^ *Sh*ER resulted in the decreased activity of CATs (87%). For all treatments with CHLA (0.005, 0.01, 0.1, 0.2, and 0.3 mg mL^−1^), the activity of CATs was increased (Figure 5B(a)). The highest increase (278%) was recorded for the treatment with 0.3 mg mL^−1^ CHLA. Similarly, all applied treatments with *p*HBA increased the activity of CATs, with the exception of 0.3 mg mL^−1^ *p*HBA, which exhibited an inhibitory effect (Figure 5B(a)).

The results of spectrophotometric quantification of the total activity of CAT in *Ch. murale* seedlings for treatments with *Sh*ER, CHLA, and *p*HBA showed a similar trend of activity (Figure 5B(a,b)), as obtained in the in-gel assays. A statistically significant increase in the activity of CAT occurred for all treatments with *Sh*ER, CHLA, and *p*HBA, with respect to the control, where 13.4 U mg^−1^ CAT activity was recorded. Treatments with 0.01 mg mL^−1^ *Sh*ER and 0.3 mg mL^−1^ CHLA resulted in the most pronounced increase in CAT activity (Figure 5B(b)).

Electrophoretic in-gel detection revealed two isoforms of POX (POX1 and POX2) in *Ch. murale* seedlings, regardless of the treatment with *Sh*ER, CHLA, and *p*HBA. Two isoforms of POX in *Ch. murale* were also observed by Bogdanović et al. [86]. The in-gel activity of the POX2 isoform was proven to be higher than that of POX1 for all treatments and in control seedlings. The activity of total POX in the control group of seedlings, as revealed by the spectrophotometric approach, was considered as 100% activity. For all *Sh*ER treatments, there was a decrease in the activity of POX (ranging from 96% to 54%). All of the treatments with CHLA resulted in the increased activity of POX with respect to the control (100%). The highest increase in POX activity (248% and 261%) was recorded at 0.005 and 0.3 mg mL^−1^ CHLA, respectively. POX activity was generally increased by treatment with *p*HBA, with the exception of 0.005 mg mL^−1^ *p*HBA, where a slight decrease (88%) was recorded (Figure 5C(a)). The results of spectrophotometric quantification of total POX activity in *Ch. murale* seedlings revealed that only the treatment with 2 mg mL^−1^ *Sh*ER resulted in a change in POX activity, inducing inhibition. It was shown that *p*HBA treatments had no significant effect on POX activity. On the other hand, treatments with 0.005, 0.01, and 0.3 mg mL^−1^ CHLA increased POX activity with respect to the control, where it was 0.7 U mg^−1^ (Figure 5C(b)). Politycka [87] showed that phenolic acids (*p*-coumaric and ferulic acids) increased the activity of POX in the cell walls of cucumber (*Cucumis sativus*) roots due to increased hydrogen peroxide production.

Electrophoretic in-gel detection revealed the presence of three isoforms of SOD (SOD1, SOD2, and SOD3) in all analyzed samples of *Ch. murale* seedlings. This was in accordance with a previous study that described changes in the activities of antioxidant enzymes during germination of *Ch. murale* [86]. The activity of the SOD3 isoform was generally higher than that of the SOD1 and SOD2 isoforms. Increased SOD activity was recorded for treatments with 0.01 and 1 mg/mL mg mL^−1^ *Sh*ER (158% and 109%, respectively), while other applied *Sh*ER concentrations reduced the total SOD activity in comparison to the control (Figure 5D(a)). All of the treatments with CHLA resulted in a significant increase of total SOD activity, with 0.3 mg mL^−1^ CHLA being the most efficient treatment (207%). Treatment with *p*HBA generally resulted in increased SOD activity, with the exception of treatment with 0.3 mg mL^−1^ *p*HBA, which displayed an inhibitory effect (92%) (Figure 5D(a)). Total activity of SOD in *Ch. murale* seedlings, as measured spectrophotometrically, was not statistically significantly changed at the applied *p*HBA concentrations (Figure 5D(b)) and was at the level in the control samples (9 U mg^−1^). The lowest applied concentration of *Sh*ER (0.01 mg mL^−1^ ) resulted in a statistically significant increase in SOD activity compared to the control, but a further increase in *Sh*ER concentration significantly reduced the activity of this antioxidant enzyme. Treatments with 0.005 and 0.3 mg mL^−1^ CHLA led to statistically significant increases in SOD activity in *Ch. murale* seedlings, while a reduction was observed for the treatment with 0.1 mg mL^−1^ CHLA (Figure 5D(b)).

Recent studies demonstrated that extracts of certain plant species can increase the activity of antioxidant enzymes in treated plants, thus contributing to better growth of *Chenopodiastrum quinoa*, an economically important crop [88]. Our results showed that *Sh*ER affected the biochemical processes in *Ch. murale* seedlings and disturbed the normal activity model of antioxidant enzymes (CAT, POX, and SOD). There was an increase in the activity of CAT in seedlings treated with *Sh*ER, CHLA, and *p*HBA, whereas the activity of POX was increased for treatments with 0.005 and 0.3 mg mL^−1^ CHLA. Similarly, SOD activity generally decreased for treatments with the tested phytotoxic agents (*Sh*ER, *p*HBA, and CHLA). The results indicated that CATs were the major enzymes responsible for the removal of ROS, which were generated during the exposure of *Ch. murale* seedlings to the extract of *S. halepense* rhizomes.

It has been reported that the phytotoxicity of CHLA usually occurs via the inhibition of primary root formation, disruption of seedling membrane integrity by induction of lipid peroxidation, and by altering the soluble protein content and antioxidant enzyme activities in *Festuca arundinacea* [89]. CHLA treatment increased the activity of SOD in *F. arundinacea*, and this effect was pH dependent [89]. Recent research [90] showed that exogenously applied CHLA alleviated oxidative damage in plant tissues by regulating water status, antioxidant capacity, redox balance, and fatty acid composition. On the other hand, the allelopathic effects of *p*HBA on various plants might be mediated by changes in rhizosphere microbial communities [91,92], alterations in photosynthetic characteristics [14], as well as by inhibition of root growth via regulating ROS accumulation [93]. In *Pogostemon cablin*, *p*HBA stress inhibited root biomass accumulation, induced excessive hydrogen peroxide accumulation and lipid peroxidation, and activated most antioxidant enzymes [94].

The pronounced phytotoxic response observed in our study, particularly against *Ch. murale* and *D. stramonium*, might have important implications for weed control in the field. Recent systematic reviews have emphasized that while many allelopathic effects have been demonstrated under laboratory and greenhouse conditions, only some of these have been validated under field conditions. Several studies have reported more than 50% suppression of weed growth following the application of allelochemical extracts as a mulch or soil additive [95]. In addition, members of the family Poaceae, including *S. halepense*, have shown allelopathic suppression of competing species under different soil types and environmental conditions, with significant reductions in both germination and seedling biomass [96]. These findings suggest that *Sh*ER, rich in *p*HBA and CHLA, could have practical value as a natural herbicide when applied in appropriate formulations and dosages. Consistent with this, Kato-Noguchi [97] demonstrated that allelochemical extracts isolated and identified from plant biomass exhibited effective phytotoxic activity in pre-field and open soil conditions, indicating their suitability for bioherbicidal formulations that can be applied as sprays, soil amendments, or post-harvest treatments. The species-specific efficacy observed in our study is aligned with the current ecological perspective that emphasizes the need for allelopathic agents capable of selective regulation of weed community dynamics and enhancing crop competitiveness in agroecosystems [24]. In addition, the utilization of *S. halepense* rhizome biomass as a feedstock for the development of bioherbicides represents a double ecological benefit—weed control and biomass exploitation.

## 3. Materials and Methods

### 3.1. Plant Material

Rhizomes of *Sorghum halepense* L. were collected in 2020 from the Lipovička Forest area, Belgrade (Serbia). Seeds of *Chenopodiastrum murale* L. were obtained from the Institute of Experimental Botany of the Czech Academy of Sciences, Prague, the Czech Republic. After *Ch. murale* plants were grown under greenhouse conditions at the Institute for Biological Research “Siniša Stanković,” the National Institute of the Republic of Serbia, University of Belgrade, Serbia, seeds were collected in 1998 and further used for the experiments. *Datura stramonium* L. and *Amaranthus retroflexus* L. seeds were collected in 2020 from the Zemun Polje area, Belgrade, Serbia.

### 3.2. LC-ESI-QTOF-MS/MS Non-Targeted Metabolomics of S. halepense Methanol Extract

The analyses were carried out using an Agilent 1290 Infinity UHPLC system coupled with quadrupole time-of-flight mass spectrometry (6530C QTOF-MS) from Agilent Technologies, Inc. (Santa Clara, CA, USA). The chromatographic separation was performed at 40 °C on a Zorbax C18 column (2.1 × 50 mm, 1.8 µm particle size) from Agilent Technologies, Inc. (CA, USA). The mobile phase consisted of a mixture of (A) ultrapure water + 0.1% HCOOH (MS grade) and (B) acetonitrile MS grade + 0.1% HCOOH (MS grade). The flow rate was kept constant at 0.3 mL min^−1^, and the injection volume was 5 µL. The gradient elution program and all MS parameters and ion source settings were previously described by Kostić et al. [98].

Agilent MassHunter software (version B.09.00; Agilent Technologies, Santa Clara, CA, USA) was used for data evaluation and analysis. Metabolites were identified based on their monoisotopic mass and MS/MS fragmentation and confirmed using previously reported data on *Sorghum* species [41,44,51] and other species from the family Poaceae [47,50]. Accurate masses of components were calculated using ChemDraw software (version 12.0, CambridgeSoft, Cambridge, MA, USA). The CAS SciFinder-n database was used to search for chemical compounds by formulas and structures (https://scifinder-n.cas.org). For the evaluation of MS data, R Studio (version 4.3.1) software (enviPick and xcms R packages) was used [99].

### 3.3. Preparation of Dry Extract of S. halepense Rhizomes and Its UHPLC/DAD/(–)HESI–MS^2^ Metabolic Profiling

The rhizomes of *S. halepense* were cleaned, dried, and cut using a blender. After that, they were ground in liquid nitrogen and extracted with 99.8% methanol (AppliChem, Cheshire, CT, USA) in a plastic foil-sealed Erlenmeyer flask (500 mL) with vigorous shaking and left overnight at room temperature. The solution was filtered through filter paper and then dried using a rotary evaporator (Eppendorf concentrator 5301, Hamburg, Germany). The crude extract was stored at −20 °C until use.

Quantification of targeted compounds was performed using a Dionex Ultimate 3000 Ultra-UHPLC system connected to a triple-quadrupole (QqQ) mass spectrometer (TSQ Quantum Access Max, Thermo Fisher Scientific, Bremen, Germany). A Syncronis C18 analytical column (100 × 2.1 mm) with 1.7 µm particle size (Thermo Fisher Scientific, Bremen, Germany) was used for the chromatographic separation of the methanol extract of *S. halepense* rhizomes. The flow rate and the composition of the mobile phases, as well as the gradient elution program, were set according to parameters previously described by Gašić et al. [100]. The mass detector was equipped with an HESI source operated in negative ionization mode, using settings previously described by Banjanac et al. [101].

The selected reaction monitoring (SRM) mode of the instrument was used for the quantification of the targeted compounds in samples. Compounds of interest were identified based on their UV, MS, and MS/MS spectra and comparison with standards. Calibration curves revealed good linearity, with *r*^2^ values exceeding 0.99 (peak areas vs. concentration). The total amount of each compound was evaluated by calculation of the peak area and is expressed as ng mg^−1^ DW.

### 3.4. Preparation of Working Solutions of S. halepense Extract, pHBA, and CHLA

The concentration of the *Sh*ER stock solution was 10 mg mL^−1^. It was further diluted with deionized water containing nystatin (500 mg L^−1^) to reach *Sh*ER concentrations of 5, 2, 1, 0.1, and 0.01 mg mL^−1^. The stock solutions of *p*HBA and CHLA (Sigma, Aldrich, Germany, both), each at a concentration of 10 mg mL^−1^, were diluted with deionized water containing nystatin (500 mg L^−1^), to prepare 0.3, 0.2, 0.1, 0.01, and 0.005 mg mL^−1^ solutions. Deionized water with nystatin was used as a control.

### 3.5. Sorghum halepense Phytotoxicity Bioassay Against Selected Weeds

#### 3.5.1. Seed Germination and Seedling Growth of *A. retroflexus*, *D. stramonium*, and *Ch. murale*

To investigate the potential bioherbicidal properties of the methanol extract of *S. halepense* rhizome, we initially recorded its effects on the germination of *A. retroflexus*, *D. stramonium,* and *Ch. murale* seeds. In parallel, we monitored the effects of *p*HBA and CHLA at different concentrations. The germination percentage was recorded every day to track the germination dynamics.

Seeds of *A. retroflexus*, *D. stramonium*, and *Ch. murale* were placed on Petri dishes (9 cm diameter), each containing a single layer of filter paper moistened with 4 mL of *Sh*ER, *p*HBA, or CHLA solution. Each biological replicate consisted of 20–30 seeds, and all concentrations of *Sh*ER, *p*HBA, and CHLA were tested in triplicate. For *A. retroflexus* seeds, the highest concentration of *p*HBA and CHLA tested was 0.2 mg mL^−1^. Seeds were germinated under a 16 h/8 h light/dark regime with a photon flux density of 70 µmol m^−2^ s^−1^ and at a temperature of 25 ± 2 °C. Germination of *A. retroflexus* seeds was recorded on a daily basis for 7 days and *D. stramonium* for 10 days, while *Ch. murale* seed germination was counted for 11 days. The phytotoxic activity of *Sh*ER and its major phenolic constituents (*p*HBA and CHLA) was further evaluated by recording the cotyledon, hypocotyl, and root lengths of all tested seedlings at the end of the experiments.

#### 3.5.2. Determination of Catalase, Peroxidase, and Superoxide Dismutase Activities in *Chenopodiastrum murale* L. Seedlings

Oxidative stress was evaluated by analyzing the activities of CAT (EC 1.11.1.6), POX (EC 1.11.1.7), and superoxide dismutase (SOD, EC 1.15.1.1) in *Ch. murale* seedlings treated with *Sh*ER, *p*HBA, and CHLA solutions for 11 days. At the end of the experiment, seedlings were rinsed in liquid nitrogen (LN) and mechanically ground into a fine powder. A protein extraction buffer (PEB), consisting of 50 mM Tris-HCl, 1 mM EDTA, 30% glycerol, 1.5% (*w*/*v*) polyvinylpolypyrrolidone, 10 mM dithiothreitol, and 1 mM phenylmethylsulfonyl fluoride, was added to samples at a ratio of 1:3 (sample mass/buffer volume). The mixtures were transferred to Eppendorf tubes (2 mL) and centrifuged at 10,000× *g* for 10 min at 4 °C. After centrifugation, the supernatants were collected in new Eppendorf tubes. Total protein concentration was accurately determined using the Qubit protein assay kit and a Qubit^®^ 3.0 fluorometer (Invitrogen, Thermo Fisher Scientific, Waltham, MA, USA, both). Samples of proteins were stored in a freezer at −80 °C until use.

Separation of isoenzymes of CAT, POX, and SOD was performed in polyacrylamide gel by non-denaturing electrophoresis (Native PAGE), as previously reported [78]. The modification of the protocol included the use of 4-chloro-ɑ-naphthol (0.2%) and H_2_O_2_ (0.1%) solutions in 50 mM K-phosphate buffer (pH 6.5) to visualize POX activity. The gels were photographed using the Transilluminator Gel-Doc^TM^ EQ system (Life Science Research, Bio-Rad Co., Hercules, CA, USA). Original gels are presented in Figure S5. The activities of total CAT, POX, and SOD were quantified spectrophotometrically (Agilent 8453, Life Sciences, Santa Clara, CA, USA).

### 3.6. Statistical Analyses

The computer package Statgraphics Centurion XVI was used for statistical analyses. One-way analysis of variance (ANOVA) was used to compare numerical results. Fisher’s LSD (Least Significant Difference) test with a significance level of *p* ≤ 0.05 was used to determine the statistical significance of differences between means. Results were plotted graphically using the Microsoft Office Excel computer program. Different letters in graphs indicate statistically significant differences.

For HCA, the input variables were scaled between min and max values for each of the metabolites independently. HCA was performed based on Pearson’s method of cluster agglomeration, adopting Morpheus software version 5.5.1 (https://software.broadinstitute.org/morpheus (accessed on 21 May 2025)). PCA was performed based on Euclidean distances with cluster agglomeration using Ward’s [102] minimum variance method, adopting Past 4 software, version 4.13 [103].

## 4. Conclusions

The innovative use of an invasive species, *Sorghum halepense*, as a source of bioherbicidal compounds represents a novel approach to sustainable weed management. The methanol extract of *S. halepense* rhizomes may offer an alternative approach for weed control, potentially highly efficient in suppressing noxious weeds from the families Amaranthaceae and Solanaceae. However, further research is needed to uncover the exact mechanisms of action of the major allelopathic compounds from the rhizomes of *S. halepense* against different weed and crop species, clarify their roles, evaluate potential toxic effects on non-target organisms, and validate the effects under field conditions. Such insights will support the development of biocontrol strategies and agricultural practices aimed at the sustainable exploitation of this weed’s allelopathic properties and the formulation of environmentally friendly bioherbicides.

## Figures and Tables

**Table 1:** LC-ESI-QTOF-MS/MS data on metabolites identified in different parts of *Sorghum halepense*.

No	Compound Name	*t*_R_, min	Molecular Formula, [M–H]^−^	Calculated Mass, [M–H]^−^	Exact Mass, [M–H]^−^	Δ mDa	MS^2^ Fragments, (% Base Peak)	I	L	R	Previously Reported in *Sorghum* or *Poaceae*
***Hydroxybenzoic acid derivatives***											
**1**	**Hydroxybenzoyl hexoside 1**	2.15	C_13_H_15_O_8_^−^	299.07724	299.07568	1.56	137.02179 *(100)	✚	✚	✚	[41]
**2**	**Vanilloyl hexoside 1**	2.96	C_14_H_17_O_9_^−^	329.08781	329.08659	1.22	108.01971(47), **121.02713**(100), 123.04285(17), 152.00925(30), 167.03219(17)	✚	–	✚	[42]
**3**	**Dihydroxybenzoyl hexoside**	3.10	C_13_H_15_O_9_^−^	315.07216	315.07099	1.17	**108.01974**(100), 152.00914(60)	✚	✚	✚	[43]
**4**	**Dihydroxybenzoic acid 1**	3.30	C_7_H_5_O_4_^−^	153.01933	153.01826	1.07	**108.02016**(100)	✚	✚	✚	[44]
**5**	**Hydroxybenzoyl hexoside 2**	3.36	C_13_H_15_O_8_^−^	299.07724	299.07578	1.46	101.02286(27), 107.04863(4), 113.02221(31), 121.02907(9), **137.02210**(100), 151.03775(17), 179.03278(26)	✚	✚	✚	[41]
**6**	**Syringoyl hexoside**	4.04	C_15_H_19_O_10_^−^	359.09837	359.09774	0.63	101.02225(22), **107.04837**(100), 113.02147(17), 135.04300(4), 151.03719(35), 179.03365(6), 197.04271(6)	✚	✚	✚	[45]
**7**	**Vanilloyl hexoside 2**	4.25	C_14_H_17_O_9_^−^	329.08781	329.08660	1.20	**108.01940**(100), 123.04257(39), 152.00889(73), 167.0322(33)	✚	✚	✚	[42]
**8**	**Hydroxybenzoyl hexoside 3**	4.38	C_13_H_15_O_8_^−^	299.07724	299.07604	1.20	101.02245(34), 107.04782(5), 113.02153(35), 121.02782(10), **137.02172**(100), 151.03701(18), 179.03197(46)	✚	✚	✚	[41]
**9**	**Hydroxybenzoic acid**	5.66	C_7_H_5_O_3_^−^	137.02442	137.02259	1.83	NA	✚	✚	✚	[46]
**10**	**Hydroxybenzoyl-sucrose**	6.13	C_19_H_25_O_13_^−^	461.13007	461.13194	−1.88	101.02215(4), 113.02155(7), **137.02175**(100), 179.03186(3), 191.03350(4), 239.05458(5), 281.06547(15)	✚	✚	✚	[47]
**11**	**Benzoyl-sucrose**	6.20	C_19_H_25_O_12_^−^	445.13515	445.13540	−0.25	101.02213(100), **107.04798**(100), 113.02225(77), 153.08965(20), 159.02814(15), 161.04358(52), 401.14409(5)	–	✚	✚	[47]
**12**	**Dihydroxybenzoic acid 2**	6.40	C_7_H_5_O_4_^−^	153.01933	153.01742	1.91	**109.02747**(100), 135.00666(21)	✚	✚	✚	[44]
**13**	**Vanilloyl-hydroxybenzoyl-pentosyl hexoside**	6.47	C_27_H_31_O_16_^−^	611.16124	611.16848	−7.24	123.04236(10), 137.02159(22), 149.02245(7), 281.06386(50), 405.11637(23), 431.09596(3), **449.10662**(100)	✚	✚	✚	[47]
***Hydroxycinnamic acid derivatives***											
**14**	**3-*O*-Caffeoylquinic acid 1**	4.92	C_16_H_17_O_9_^−^	353.08781	353.08659	1.22	135.04252(91), 179.03232(39), **191.05416**(100)	✚	–	–	[44]
**15**	**3-*O*-Caffeoylquinic acid 2**	5.66	C_16_H_17_O_9_^−^	353.08781	353.08702	0.79	135.04364(87), 179.03342(41), **191.05464**(100)	✚	✚	✚	[44]
**16**	**5-*O*-Caffeoylquinic acid**	6.60	C_16_H_17_O_9_^−^	353.08781	353.08714	0.66	**191.05469**(100)	✚	✚	✚	[44]
**17**	**3-*O*-Feruloylquinic acid**	6.73	C_17_H_19_O_9_^−^	367.10346	367.10263	0.82	117.03230(9), **134.03467**(100), 149.05846(5), 193.04848(41)	✚	✚	✚	[43]
**18**	**Methyl 5-(6″-sinapoyl-hexosyl)-*O*-caffeoylquinate**	6.74	C_34_H_39_O_18_^−^	735.21366	735.21953	−5.86	134.03475(6), 173.04301(7), 191.05286(5), **193.04842**(100), 367.10189(42)	✚	✚	✚	**New**
**19**	**5-(6″-Sinapoyl-hexosyl)-*O*-caffeoylquinic acid**	6.74	C_33_H_37_O_18_^−^	721.19805	721.20149	−3.44	134.03512(6), 173.04347(31), 179.03236(18), 191.05451(17), **193.04852**(100), 353.08558(27), 367.10172(46)	✚	✚	✚	**New**
**20**	**Feruloyl hexoside**	7.00	C_16_H_19_O_9_^−^	355.10346	355.10025	3.21	**134.03437**(100), 150.05370(45), 160.01174(18), 175.03891(23), 178.02458(69), 191.05595(20), 193.05380(57)	✚	✚	✚	[48]
**21**	**3-*O*-*p*-Coumaroylquinic acid**	7.14	C_16_H_17_O_8_^−^	337.09289	337.09194	0.95	111.04307(9), 119.04809(25), 163.03799(20), 173.04338(5), **191.05405**(100)	✚	✚	–	[49]
**22**	**5-*O*-Caffeoylshikimic acid**	7.34	C_16_H_15_O_8_^−^	335.07724	335.07999	−2.75	119.04747(3), 123.00756(3), **135.04220**(100), 161.02286(22), 179.03177(34)	✚	✚	–	[44]
**23**	**4-*O*-Feruloylquinic acid**	7.48	C_17_H_19_O_9_^−^	367.10346	367.10271	0.75	117.03300(5), 134.03563(44), 149.05952(3), 173.04398(39), **191.05467**(100), 193.05006(22)	✚	✚	✚	[50]
**24**	**1-*O*-Coumaroyl-glycerol**	7.61	C_12_H_13_O_5_^−^	237.07685	237.07532	1.53	117.03245(95), 119.04838(34), **145.02727**(100)	✚	✚	✚	[51]
**25**	***p*-Coumaric acid**	7.61	C_9_H_7_O_3_^−^	163.04007	163.03839	1.68	117.03245(7), **119.04793**(100)	✚	✚	✚	[52]
**26**	**1-*O*-Coumaroyl-threonic acid**	8.08	C_13_H_13_O_7_^−^	281.06668	281.06817	−1.49	119.04865(38), **145.02815**(100), 163.03882(3)	✚	–	✚	**New**
**27**	**1,3-*O*-Dicaffeoylglycerol**	9.57	C_21_H_19_O_9_^−^	415.10346	415.10681	−3.35	133.02686(8), 135.04281(91), **161.02224**(100), 179.03278(72), 235.05948(4), 253.06927(82)	✚	–	–	[51]
**28**	**1,3-*O*-Coumaroyl-caffeoyl-glycerol**	10.17	C_21_H_19_O_8_^−^	399.10854	399.11036	−1.82	119.04860(83), 135.04341(43), 161.02391(59), **163.03862**(100), 179.03343(23), 235.05963(9), 253.07023(38)	✚	–	✚	[51]
**29**	**1,3-*O*-Feruloyl-caffeoyl-glycerol**	10.24	C_22_H_21_O_9_^−^	429.11911	429.12218	−3.08	**134.03469**(100), 149.05746(12), 161.02354(54), 179.03264(21), 193.04838(95), 235.05871(13), 253.06913(38)	–	–	✚	[51]
**30**	**Diferuloyl-sucrose tri-acetyl ester**	10.71	C_38_H_43_O_20_^−^	819.23480	819.24395	−9.15	**175.03719**(100), 193.04834(11), 345.22234(5), 601.17274(5), 643.18493(5), 759.20662(9), 777.22535(13)	–	–	✚	[47]
**31**	**1,3-*O*-Dicoumaroyl-glycerol**	10.78	C_21_H_19_O_7_^−^	383.11363	383.11644	−2.81	117.03274(8), **119.04859**(100), 145.02787(63), 163.03835(96)), 219.06400(5), 237.07500(4)	✚	✚	✚	[51]
**32**	**1,3-*O*-Coumaroyl-feruloyl-glycerol**	10.85	C_22_H_21_O_8_^−^	413.12419	413.12638	−2.19	119.04865(67), **134.03559**(100), 145.02800(43), 160.01616(15), 163.03853(65), 175.03858(23), 193.04918(59)	✚	✚	✚	[51]
**33**	**1,3-*O*-Diferuloyl-glycerol**	10.98	C_23_H_23_O_9_^−^	443.13476	443.13512	−0.37	134.03582(99), 135.04012(12), 149.05882(20), 160.01751(16), 175.03815(35), **193.04941**(100)	✚	✚	✚	[51]
**34**	**Diferuloyl-sucrose tetra-acetyl ester**	11.66	C_40_H_45_O_21_^−^	861.24588	861.24459	1.29	**175.03794**(100), 193.04839(9), 625.17540(4), 643.18719(5), 685.19914(4), 801.22476(15), 819.2327(14)	✚	✚	✚	[47]
***Flavonoid glycosides***											
**35**	**Quercetin 3,4′-di-*O*-hexoside**	6.80	C_27_H_29_O_17_^−^	625.14051	625.14821	−7.71	166.04827(28), 191.05588(9), 210.04103(14), 211.02269(68), 300.02160(16), 301.03319(54), **463.08475**(100)	✚	–	–	[51]
**36**	**Luteolin 7-*O*-(2″-pentosyl)-hexoside**	7.27	C_26_H_27_O_15_^−^	579.13502	579.13964	−4.63	369.05905(85), 399.0697(95), 429.08014(30), 441.08052(23), **459.09156**(100), 471.09038(8), 489.10134(43)	✚	✚	–	[53]
**37**	**Apigenin 6-*C*-hexoside−8-*C*-pentoside**	7.54	C_26_H_27_O_14_^−^	563.14010	563.14763	−7.52	149.04288(22), **353.06485**(100), 383.07530(77), 425.08417(19), 443.09686(46), 473.10771(49), 503.11826(27)	✚	✚	✚	[43]
**38**	**Luteolin 3′,7-di-*O*-hexoside**	7.69	C_27_H_29_O_16_^−^	609.14569	609.15500	−9.31	147.02457(7), 175.09716(12), 191.05255(84), 284.03316(18), **285.03694**(100), 286.04299(22), 447.09260(68)	–	✚	–	[54]
**39**	**Eriodictyol 7-*O*-hexoside**	8.08	C_21_H_21_O_11_^−^	449.10894	449.10916	−0.22	135.04244(16), 151.00178(46), 152.00506(6), 217.10833(11), 286.03948(81), **287.04418**(100)	✚	–	–	[51]
**40**	**Quercetin 3-*O*-hexoside**	8.08	C_21_H_19_O_12_^−^	463.08778	463.09585	−8.07	151.00124(4), 161.02153(3), 178.99940(3), 255.02771(6), 271.02293(12), **300.02573**(100), 301.03248(49)	✚	–	–	[55]
**41**	**Luteolin 7-*O*-hexoside**	8.12	C_21_H_19_O_11_^−^	447.09329	447.09491	−1.63	284.03043(39), **285.03801**(100), 286.04177(19)	✚	✚	–	[51]
**42**	**Chrysoerol 6-*C*-hexoside**	8.15	C_22_H_21_O_11_^−^	461.10842	461.11514	−6.72	109.02603(29), 131.03546(27), 151.03529(26), 298.04661(73), 299.05206(18), 313.06695(26), **341.06476**(100)	✚	✚	✚	[56]
**43**	**Chrysoerol 7-*O*-(6″-rhamnosyl)-hexoside**	8.62	C_28_H_31_O_15_^−^	607.16633	607.17486	−8.53	284.03045(15), **299.05329**(100)	✚	–	–	**New**
**44**	**Luteolin 7-*O*-(6″-caffeoyl)-hexoside**	9.10	C_30_H_25_O_14_^−^	609.12445	609.12966	−5.20	161.02202(7), 179.03113(4), **285.03821**(100), 323.07656(8), 447.08988(5)	–	✚	–	**New**
**45**	**Tricin 4′-*O*-*(erythro*-guaiacylglyceryl) ether**	10.65	C_27_H_25_O_11_^−^	525.14024	525.14179	−1.55	**165.05354**(100), 195.06397(20), 299.01771(31), 313.03283(11), 314.04096(87), 329.06420(94)	✚	✚	–	[57]
***Flavonoid aglycones***											
**46**	**Luteolin**	9.77	C_15_H_9_O_6_^−^	285.04046	285.04093	−0.47	107.01180(15), **133.02738**(100), 149.02202(12), 151.00143(30), 175.03779(14), 199.03763(11), 217.04828(7)	✚	✚	✚	[51]
**47**	**Quercetin**	9.84	C_15_H_9_O_7_^−^	301.03483	301.04473	−9.90	107.01242(38), 121.02829(41), **151.00186**(100), 178.99758(13), 215.03478(15), 243.02878(25), 257.04214(15)	✚	–	✚	[44]
**48**	**Apigenin**	10.45	C_15_H_9_O_5_^−^	269.04555	269.04626	−0.72	107.01209(38), **117.03234**(100), 121.02679(8), 149.02139(25), 151.00092(55), 227.03471(5)	–	✚	✚	[44]
**49**	**Tricin**	10.58	C_17_H_13_O_7_^−^	329.06668	329.06783	−1.15	161.02155(11), 227.03264(18), 243.02701(5), 271.02265(44), **299.01787**(100), 314.04066(14)	✚	✚	✚	[44]
**50**	**Chrysoerol**	10.65	C_16_H_11_O_6_^−^	299.05611	299.05692	−0.81	107.01199(4), 151.00100(5), 227.03333(5), 256.03499(67), **284.02993**(100)	–	✚	✚	[51]
***Fatty acids***											
**51**	**Trihydroxyoctadecadienoic acid 1**	10.38	C_18_H_31_O_5_^−^	327.21770	327.21612	1.58	127.11013(11), 137.09409(17), **171.10022**(100), 183.13578(27), 193.11929(8), 211.13147(96), 229.14154(28)	✚	✚	✚	[44]
**52**	**Trihydroxyoctadecenoic acid 1**	10.78	C_18_H_33_O_5_^−^	329.23335	329.23111	2.24	127.11068(20), 139.11053(55), **171.10041**(100), 183.13661(15), 193.12133(5), 211.13164(70), 229.1424(22)	✚	✚	✚	[51]
**53**	**Trihydroxyoctadecadienoic acid 2**	11.45	C_18_H_31_O_5_^−^	327.21770	327.21646	1.24	127.11043(27), 137.09476(29), 155.10512(28), **171.09991**(100), 199.13045(17), 201.11053(45), 209.1163(10)	✚	–	✚	[44]
**54**	**Trihydroxyoctadecenoic acid 2**	11.59	C_18_H_33_O_5_^−^	329.23335	329.23176	1.58	127.11083(36), 139.11058(20), 155.10565(13), **171.10051**(100), 199.12978(17), 201.11123(89), 293.2088(8)	✚	✚	✚	[51]
**55**	**Dihydroxyoctadecenoic acid 1**	13.07	C_18_H_33_O_4_^−^	313.23843	313.23540	3.03	127.11086(45), 129.08937(59), 171.10008(33), **183.13717**(100), 251.19894(26), 277.21524(32), 295.22696(58)	✚	–	✚	[51]
**56**	**Dihydroxyoctadecenoic acid 2**	13.34	C_18_H_33_O_4_^−^	313.23843	313.23702	1.42	127.07463(11), 129.09035(48), **183.13746**(100), 195.13807(7)	✚	✚	✚	[51]
**57**	**Dihydroxyoctadecenoic acid 3**	13.48	C_18_H_33_O_4_^−^	313.23843	313.23616	2.27	127.11053(30), 139.11077(4), 171.10026(22), 199.09509(9), **201.11113**(100), 277.21547(9)	✚	✚	✚	[51]
**58**	**Dihydroxyoctadecadienoic acid**	13.75	C_18_H_31_O_4_^−^	311.22278	311.22116	1.63	127.11004(19), 139.10962(27), **171.10031**(100), 185.11572(10), 197.11536(12), 201.11167(14), 211.12991(14)	✚	✚	✚	[51]
***Lignans***											
**59**	**Oryzativol A**	11.39	C_40_H_39_O_13_^−^	727.23962	727.24698	−7.36	119.04759(26), 145.02738(21), **163.03750**(100), 515.16625(8), 545.18149(61), 709.23082(4)	–	–	✚	[58]
**60**	**Oryzativol B**	13.07	C_40_H_39_O_13_^−^	727.23962	727.24193	−2.31	119.04820(24), 145.02674(30), **163.03781**(100), 581.20291(4)	–	–	✚	[58]

***t*_R_**—retention time (min); **Δ mDa**—mean mass accuracy; * MS^2^ base peaks are marked as bold in the table; **I**—inflorescence; **L**—leaf; **R**—root; **✚** stands for detected and—stands for non-detected compound; **NA**—not available. Compound **18** was identified as a methyl derivative of compound **19**. Such compounds were previously identified in eggplant [59], while in *Sorghum* they may be reported as new compounds. Compound **26** with a molecular ion at 281 *m*/*z* showed specific MS^2^ ions corresponding to coumaric acid and its fragments, while the neutral loss corresponded to the threonic acid residue (Figure S2). This compound was not previously identified in *Sorghum* but was found in *Fagus sylvatica* L. leaves [60]. Two of the identified compounds belonging to the group of hydroxycinnamic acids (**14** and **27**) were present only in samples of inflorescences, while compounds **29** and **30** were recorded exclusively in rhizomes.

## Data Availability

The original contributions presented in this study are included in the article/Supplementary Materials. Further inquiries can be directed to the corresponding author(s).

## Supplementary Materials

The following supporting information can be downloaded at: https://www.mdpi.com/article/10.3390/molecules30153060/s1, Table S1: Peak areas of identified compounds in *Sorghum halepense* flower, leaf, and root extracts; samples were analyzed in 3 biological replicates; Figure S1: Proposed structure and fragmentation pathway for compound **19** (5-(6″-sinapoyl-hexosyl)-*O*-caffeoylquinic acid); Figure S2: Proposed structure and fragmentation pathway for compound **26** (1-*O*-coumaroyl-threonic acid). Figure S3: Proposed structure and fragmentation pathway for compound **43** (chrysoerol 7-*O*-(6″-rhamnosyl)-hexoside); Figure S4: Proposed structure and fragmentation pathway for compound **44** (luteolin 7-*O*-(6″-caffeoyl)-hexoside); Figure S5: Original NATIVE-PAGE gels: in-gel determination of CAT, POX, and SOD activities.

## Abbreviations

The following abbreviations are used in this manuscript:

CAT	catalase
CHLA	chlorogenic acid
DT	days after the start of the treatment
HCA	hierarchical cluster analysis
Native PAGE	native polyacrylamide gel electrophoresis
PCA	principal component analysis
*p*HBA	*p*-hydroxybenzoic acid
POX	peroxidase
ROS	reactive oxygen species
*Sh*ER	methanol extract of *S. halepense* rhizomes
SOD	superoxide dismutase
