# Supplementary material for: Bioherbicidal Evaluation of Methanol Extract of Sorghum halepense L. Rhizome and Its Bioactive Components Against Selected Weed Species
Source: Molecules. 2025 Jul 22;30(15):3060. doi: 10.3390/molecules30153060 (PMC12348513; doi:10.3390/molecules30153060)
Supplement: Supplementary file 1 [file molecules-30-03060-s001.zip › Figure S5.pdf]

Figure S5. Original NATIVE-Page gels: in-gel determination of CAT, POX, and SOD activities.

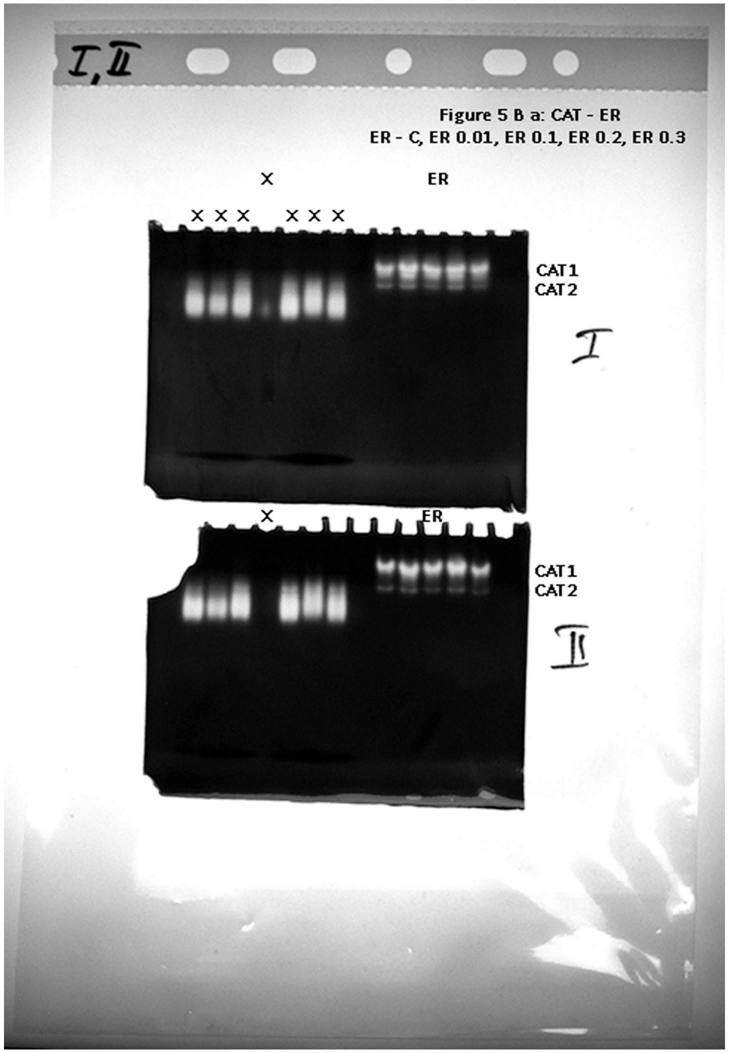

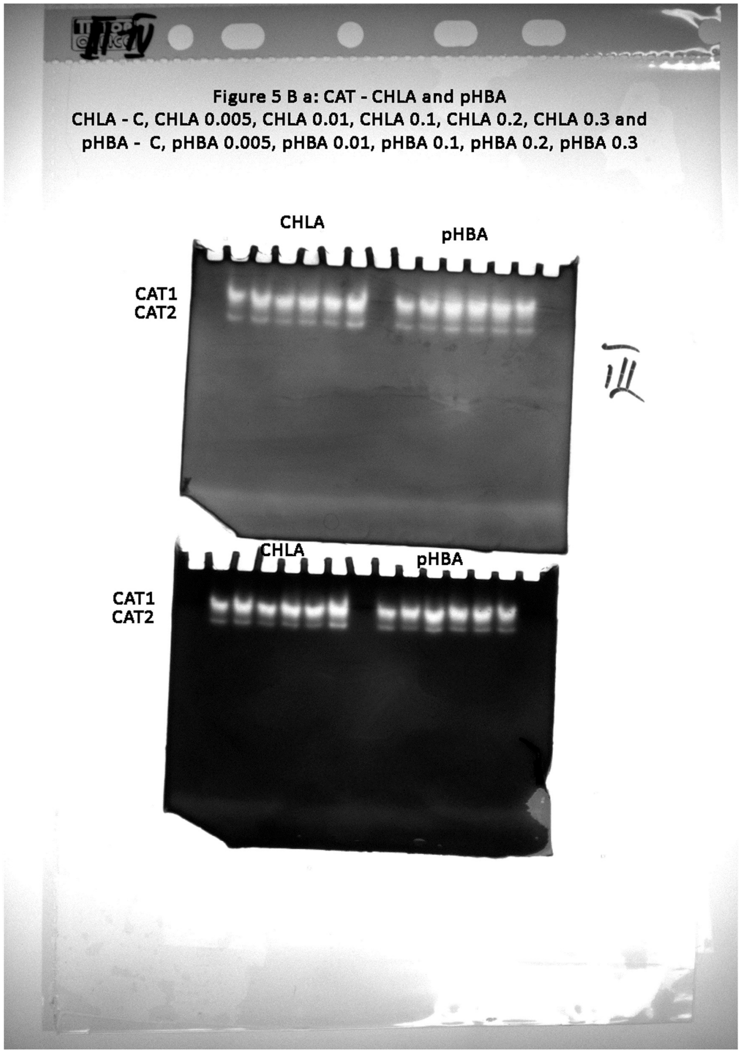

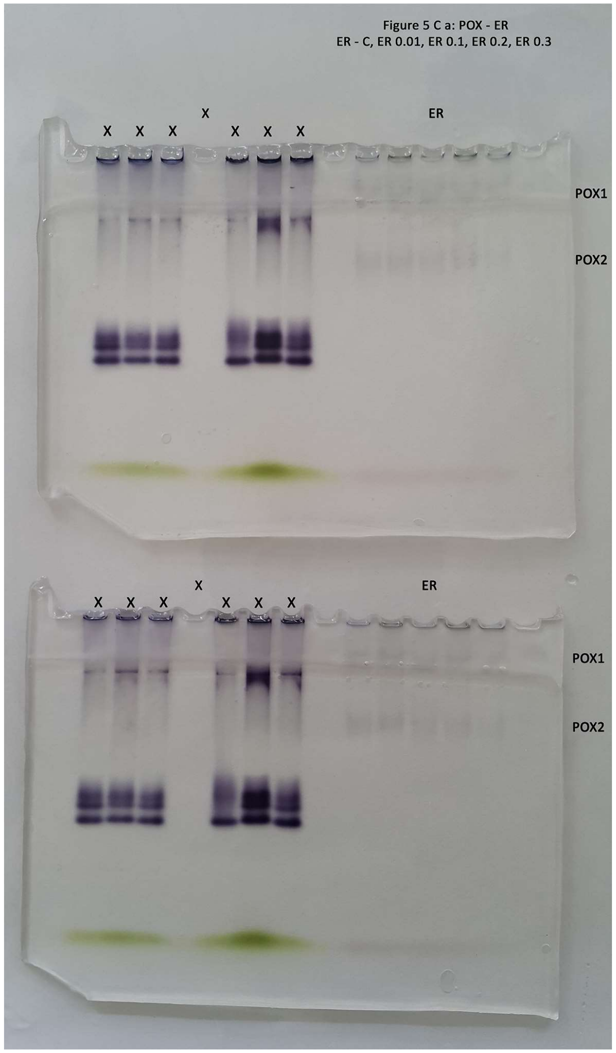

Figure 5 C a: POX - ER
ER - C, ER 0.01, ER 0.1, ER 0.2, ER 0.3

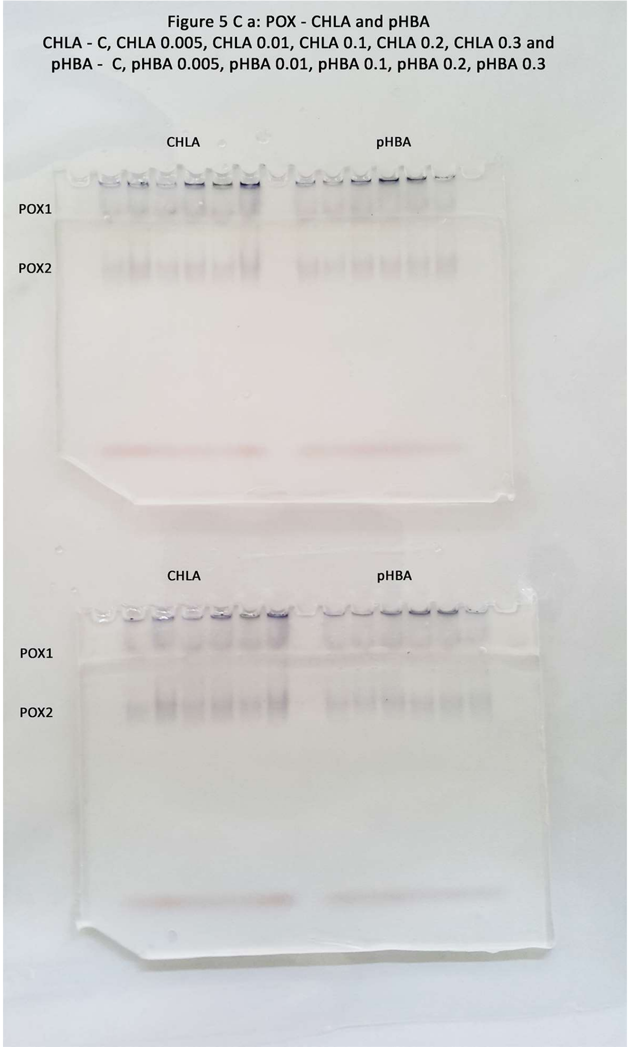

Figure 5 C a: POX - CHLA and pHBA
CHLA - C, CHLA 0.005, CHLA 0.01, CHLA 0.1, CHLA 0.2, CHLA 0.3 and
pHBA - C, pHBA 0.005, pHBA 0.01, pHBA 0.1, pHBA 0.2, pHBA 0.3

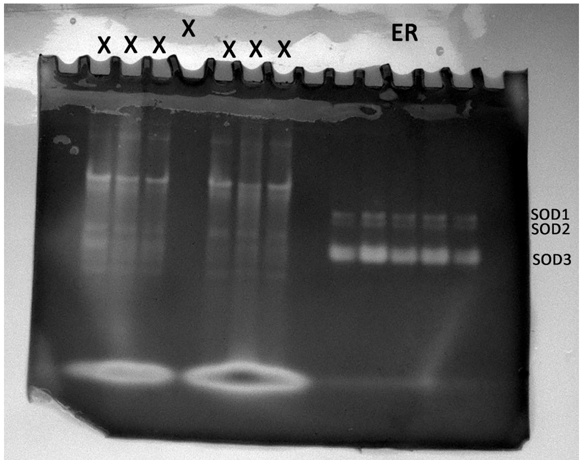

Figure 5 D a: SOD - ER
ER - C, ER 0.01, ER 0.1, ER 0.2, ER 0.3

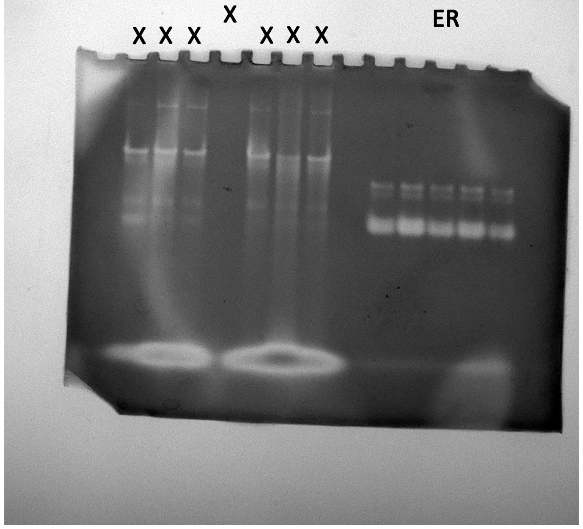

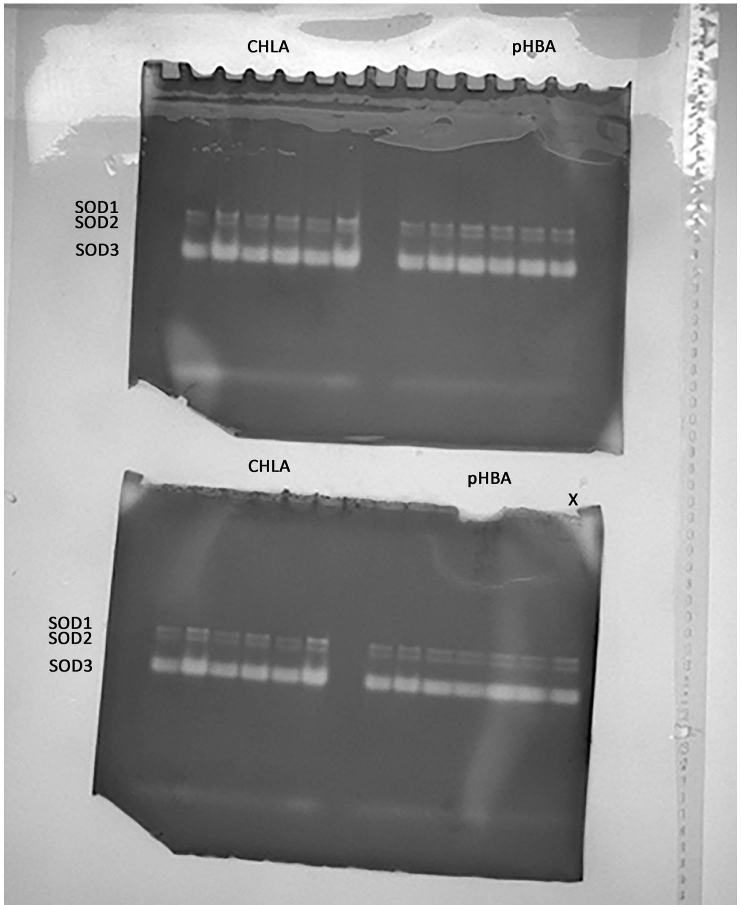

Figure 5 D a: SOD - CHLA and pHBA
CHLA - C, CHLA 0.005, CHLA 0.01, CHLA 0.1, CHLA 0.2, CHLA 0.3 and
pHBA - C, pHBA 0.005, pHBA 0.01, pHBA 0.1, pHBA 0.2, pHBA 0.3
