# Supplementary material for: Bioherbicidal Evaluation of Methanol Extract of Sorghum halepense L. Rhizome and Its Bioactive Components Against Selected Weed Species
Source: Molecules. 2025 Jul 22;30(15):3060. doi: 10.3390/molecules30153060 (PMC12348513; doi:10.3390/molecules30153060)
Supplement: Supplementary file 1 [file molecules-30-03060-s001.zip › Supplementary material (Table S1 and Figures S1-S4)-done.pdf]

Supplementary material

Bioherbicidal Evaluation of Methanol Extract of *Sorghum Halepense* L. Rhizome and Its Bioactive Components Against Selected Weed Species

Jasmina Nestorović Živković [1], Milica Simonović [2], Danijela Mišić [1], Marija Nešić [3], Vladan Jovanović [2], Uroš Gašić [1,*], Ivana Bjedov [3,*] and Slavica Dmitrović [1,*]

[1] Institute for Biological Research "Siniša Stanković", National Institute of the Republic of Serbia, University of Belgrade, Bulevar despota Stefana 142, 11108 Belgrade, Serbia; jasmina.nestorovic@ibiss.bg.ac.rs (J.N.Ž.); dmisic@ibiss.bg.ac.rs (D.M.)
[2] Institute of Pesticides and Environmental Protection, Banatska 31b, 11080 Belgrade, Serbia; milica.simonovic@pesting.org.rs (M.S.); vladanjovanovic@pesting.org.rs (V.J.)
[3] Faculty of Forestry, University of Belgrade, Kneza Višeslava 1, 11030 Belgrade, Serbia; marija.nesic@sfb.bg.ac.rs
* Correspondence: uros.gasic@ibiss.bg.ac.rs (U.G.); ivana.bjedov@sfb.bg.ac.rs (I.B.); slavica.dmitrovic@ibiss.bg.ac.rs (S.D.)

Table S1. Peak areas of identified compounds in *Sorghum halepense* flower, leaf and root extracts; samples were analyzed in 3 biological replicates.

No	Compound name	Inflorescence 1	Inflorescence 2	Inflorescence 3	Leaves1	Leaves2	Leaves3	Rhizomes 1	Rhizomes 2	Rhizomes 3
Hydroxybenzoic acid derivatives										
1	**Hydroxybenzoyl hexoside 1**	18854357	20646495	21458937	30176464	32298184	32055835	73197466	80133675	79338619
2	**Vanilloyl hexoside 1**	9980659	10011967	8025193				17587323	17802517	16769448
3	**Dihydroxybenzoyl hexoside**	6382154	2841733	4611944	36054769	48158001	56436095	14308303	23266356	23827038
4	**Dihydroxybenzoic acid 1**	54015609	56432458	61102405	24448771	23353747	23696367	26581828	21050165	22589819
5	**Hydroxybenzoyl hexoside 2**	2186631	2010562	2098596	8227435	10233473	7307692	50259939	49408360	46162030
6	**Syringoyl hexoside**	6077450	6649905	7176648	13117369	15281005	14744566	37077577	38033060	40558786
7	**Vanilloyl hexoside 2**	23256412	22554533	24080627	17709566	19457024	17942905	39609873	33193330	31128121
8	**Hydroxybenzoyl hexoside 3**	2915818	2932938	3010329	10278884	11407259	11330043	31305842	33244581	35458411
9	**Hydroxybenzoic acid**	81361097	79897214	79593977	98121981	99992906	103458846	90110751	97884633	107613460
10	**Hydroxybenzoyl-sucrose**	424167	363107	485226	1809626	2162603	2155471	6472090	7437267	7267165

**11**	**Benzoyl-sucrose**				2227622	1484659	1699410	5558585	7107443	7130618
**12**	**Dihydroxybenzoic acid 2**	11713147	11970060	13215952	136901884	160358837	165356441	41184540	41575502	42870704
**13**	**Vanilloyl-hydroxybenzoyl-pentosyl hexoside**	310236	354090	266383	8718784	6851113	8063405	10336096	18549535	15233857
Hydroxycinnamic aicd derivatives										
**14**	**3-*O*-Caffeoylquinic acid 1**	30528036	32822098	31739114						
**15**	**3-*O*-Caffeoylquinic acid 2**	48387036	43483068	53450744	18954503	22520141	24846074	1713069	1852377	2205570
**16**	**5-*O*-Caffeoylquinic acid**	118767273	118177097	119662369	65382389	78725427	73493152	56205174	65111988	70870479
**17**	**3-*O*-Feruloylquinic acid**	56342487	57097641	58545292	64923902	70962546	73534012	55207413	63685103	61337064
**18**	**Methyl 5-(6”-sinapoyl-hexosyl)-*O*-caffeoylquinate**	8452101	7933275	7772964	9258448	12433324	12642494	3246916	5116842	5160356
**19**	**5-(6”-Sinapoyl-hexosyl)-*O*-caffeoylquinic acid**	28220933	29035368	26905817	22317470	20042324	22112805	4092384	3585559	3810482
**20**	**Feruloyl hexoside**	6677848	7276506	7737620	4222068	4838475	5013254	2895298	2999693	3301814
**21**	**3-*O*-*p*-Coumaroylquinic acid**	23012356	21926474	22934834	2602011	2767372	2873309			
**22**	**5-*O*-Caffeoylshikimic acid**	2807713	2635768	2721741	6295508	4112939	4341891			
**23**	**4-*O*-Feruloylquinic acid**	25493331	25208491	23105952	16338064	15318962	17393965	11121948	11257840	11609877
**24**	**1-*O*-Coumaroyl-glycerol**	108253844	110414393	105116738	3535666	4454308	3816917	17766311	30830991	27801940
**25**	***p*-Coumaric acid**	8367724	8071673	8422749	14356244	16561292	15112778	61429370	68700400	70124649
**26**	**1-*O*-Coumaroyl-threonic acid**	9268414	9112254	12917799				482803	1586131	1854202
**27**	**1,3-*O*-Dicaffeoylglycerol**	14797963	14680408	11884224						
**28**	**1,3-*O*-Coumaroyl-caffeoyl-glycerol**	28331150	40284188	25357686				5292875	9212991	9443689
**29**	**1,3-*O*-Feruloyl-caffeoyl-glycerol**							4680249	12846804	10921587
**30**	**Diferuloyl-sucrose tri-acetyl ester**							9210776	9795200	11427387
**31**	**1,3-*O*-Dicoumaroyl-glycerol**	16797696	24034579	13555601	3286596	4398946	4739711	9428719	11871295	13184028
**32**	**1,3-*O*-Coumaroyl-feruloyl-glycerol**	107668137	118721460	101754156	12859204	12835589	9730629	28307916	65205578	44085321
**33**	**1,3-*O*-Diferuloyl-glycerol**	41988051	45422775	55214803	6147598	4583203	7711994	34024431	33431207	34848069
**34**	**Diferuloyl-sucrose tetra-acetyl ester**	4766465	7441123	4584028	2458771	4683803	4052940	11943674	18250859	17646521
Flavonoid glycosides										
**35**	**Quercetin 3,4'-di-*O*-hexoside**	8798278	8774467	8532678						
**36**	**Luteolin 7-*O*-(2"-pentosyl)-hexoside**	7982624	8141354	7558898	28103906	24152988	25615389			
**37**	**Apigenin 6-*C*-hexoside-8-*C*-pentoside**	6862180	7128254	6362628	13565228	13588090	14673674	5836361	7735407	8246758

**38**	**Luteolin 3′,7-di-*O*-hexoside**				4511906	3855130	4643247			
**39**	**Eriodictyol 7-*O*-hexoside**	5801874	4949593	5925731						
**40**	**Quercetin 3-*O*-hexoside**	7866290	7996354	6928559						
**41**	**Luteolin 7-*O*-hexoside**	12518801	12691650	12277345	31276969	29161643	31137461			
**42**	**Chrysoerol 6-*C*-hexoside**	3148400	3244090	3262730	2203868	3544606	2563065	1728253	3323896	2997062
**43**	**Chrysoerol 7-*O*-(6"-rhamnosyl)-hexoside**	1020875	1326436	1173656						
**44**	**Luteolin 7-*O*-(6"-caffeoyl)-hexoside**				8263526	6240297	8296278			
**45**	**Tricin 4′-*O*-(*erythro*-guaiacylglyceryl) ether**	2632654	2197120	2941286	7543238	9150853	10814789			
Flavonoid aglycones										
**46**	**Luteolin**	34557486	37304594	33366614	78727773	74920340	80217755	53298388	54539433	58063006
**47**	**Quercetin**	4921490	5028973	6425796						
**48**	**Apigenin**				1957162	1915878	1874593	4716688	7203644	5723493
**49**	**Tricin**	11413709	15435537	10650364	17512102	19071338	22238096	3708887	6521131	6336843
**50**	**Chrysoerol**				3114616	3358259	3216218	8807086	8399634	10477274
Fatty acids										
**51**	**Trihydroxyoctadecadienoic acid 1**	40688296	45899391	34954358	74078482	87238560	84147846	28180340	30693725	36692472
**52**	**Trihydroxyoctadecenoic acid 1**	57063952	46934804	54609842	56661944	40534672	36328879	76121610	61260895	60829066
**53**	**Trihydroxyoctadecadienoic acid 2**	60730630	13454299	39729952				35367091	25055976	14744860
**54**	**Trihydroxyoctadecenoic acid 2**	26600662	25372102	23399367	16214046	12101802	12994828	20953612	19933206	19534692
**55**	**Dihydroxyoctadecenoic acid 1**	8825946	10423637	7143246				5488936	3137069	3217485
**56**	**Dihydroxyoctadecenoic acid 2**	52321131	67041312	45314968	15368020	15007476	14874065	24991764	30976343	33256571
**57**	**Dihydroxyoctadecenoic acid 3**	35256669	40920715	25155198	14144827	18665473	14400217	21864270	28608424	32438281
**58**	**Dihydroxyoctadecadienoic acid**	16603669	21626608	19115139	10228427	10280214	10332001	16047345	17654084	19943773
Lignans										
**59**	**Oryzativol A**							2470851	6361947	5463533
**60**	**Oryzativol B**							12198133	23631261	22195123

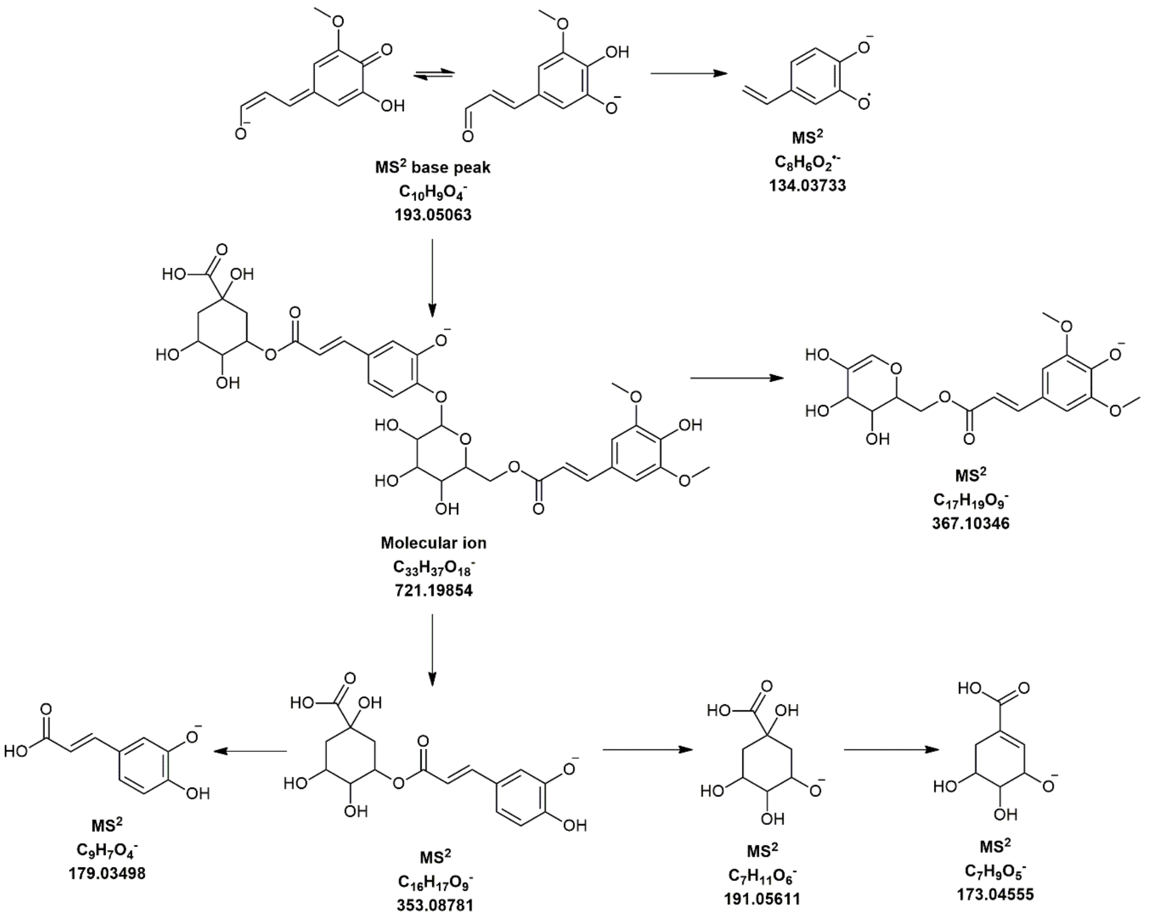

Figure S1. Proposed structure and fragmentation pathway for compound **19** (5-(6”-sinapoyl-hexosyl)-*O*-caffeoylquinic acid).

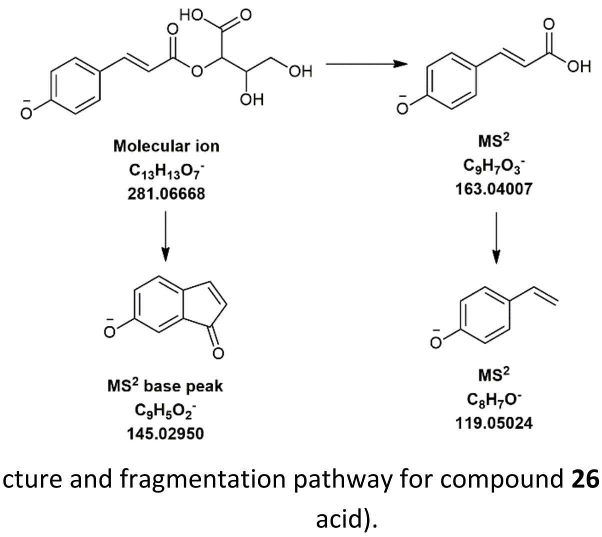

Figure S2. Proposed structure and fragmentation pathway for compound **26** (1-*O*-coumaroyl-threonic acid).

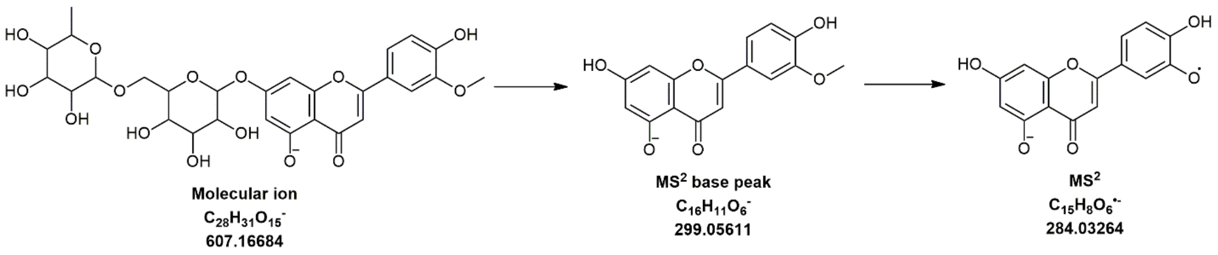

Figure S3. Proposed structure and fragmentation pathway for compound **43** (chrysoerol 7-*O*-(6"-rhamnosyl)-hexoside).

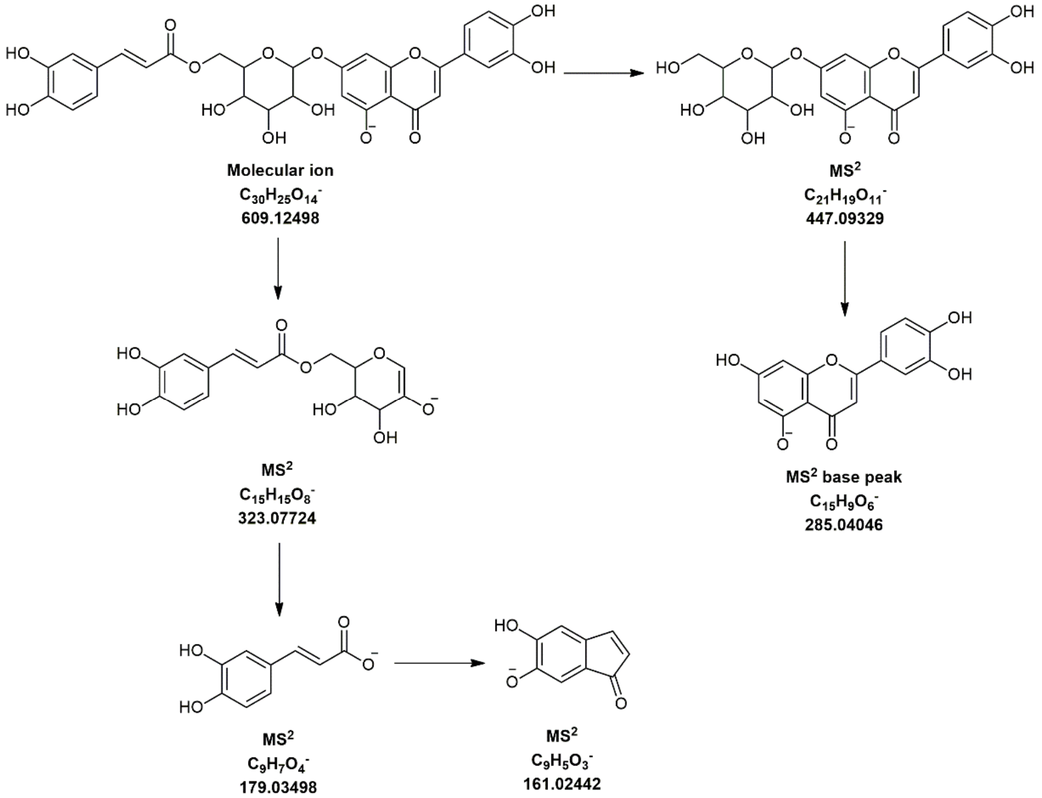

Figure S4. Proposed structure and fragmentation pathway for compound **44** (luteolin 7-*O*-(6"-caffeoyl)-hexoside).
